# BAFF-R expression as a potential biomarker associated with COVID-19 vaccine non-responsiveness in antibody-deficient patients

**DOI:** 10.1093/cei/uxag004

**Published:** 2026-01-20

**Authors:** Eleanor O’Callaghan, Adrian M Shields, Leon Chang, Michelle Umpierrez, Darren Newton, Siobhan O Burns, Alex G Richter, Gina Doody, Sinisa Savic

**Affiliations:** Leeds Institute of Rheumatic and Musculoskeletal Medicine, University of Leeds, Leeds, UK; Clinical Immunology Services, School of Infection, Inflammation and Immunology, College of Medicine and Health, University of Birmingham, Birmingham, UK; Department of Clinical Immunology, University Hospitals Birmingham National Health Service (NHS) Foundation Trust, Birmingham, UK; Leeds Institute of Rheumatic and Musculoskeletal Medicine, University of Leeds, Leeds, UK; Leeds Institute of Rheumatic and Musculoskeletal Medicine, University of Leeds, Leeds, UK; Leeds Institute of Medical Research, University of Leeds, Leeds, UK; Department of Immunology, Royal Free London National Health Service (NHS) Foundation Trust, London, UK; Institute of Immunity and Transplantation, University College London, London, UK; Clinical Immunology Services, School of Infection, Inflammation and Immunology, College of Medicine and Health, University of Birmingham, Birmingham, UK; Department of Clinical Immunology, University Hospitals Birmingham National Health Service (NHS) Foundation Trust, Birmingham, UK; Leeds Institute of Medical Research, University of Leeds, Leeds, UK; Leeds Institute of Rheumatic and Musculoskeletal Medicine, University of Leeds, Leeds, UK; Department of Clinical Immunology and Allergy, St James’s University Hospital, Leeds, UK; NIHR Leeds Biomedical Research Centre, Leeds, UK

**Keywords:** BAFF-R, antibody deficiency, SARS-CoV-2 vaccination, vaccine non-responsiveness, B-cell differentiation

## Abstract

Patients with primary and secondary antibody deficiencies exhibit variable responses to vaccination, with many failing to mount optimal immunity to SARS-CoV-2. Mechanisms underpinning vaccine non-responsiveness remain poorly defined and unpredictable. We hypothesized that B-cell-intrinsic features are associated with SARS-CoV-2 vaccine failure. Peripheral B-cells from 49 patients enrolled in the COVID-19 in Antibody Deficiency (COV-AD) study underwent a validated *in vitro* B-cell differentiation assay. We assessed plasmablast and plasma cell (PC) generation, immunoglobulin production, immunoglobulin heavy chain (IGH) repertoire diversity, and B-cell activating factor receptor (BAFF-R) expression. Vaccine non-responders displayed reduced IgA class-switched immunoglobulin production *in vitro* compared with healthy controls (HCs) and responders. Moreover, while the relative percentage of PC output was comparable between groups, the overall number of cells obtained from non-responders was reduced. Most non-responders and a subset of responders exhibited reduced BAFF-R surface expression at baseline compared with HCs, though with considerable overlap between groups. BAFF-R transcript levels partially corresponded with surface expression but varied and did not clearly distinguish response. No compensatory upregulation of alternative B-cell activating factor (BAFF) receptors or elevated serum BAFF was observed. IGH repertoire analysis revealed preserved diversity among patients. Diminished BAFF-R expression is associated with vaccine non-responsiveness and may indicate underlying B-cell-intrinsic defects. BAFF-R shows potential as a candidate biomarker that merits further validation in larger, multicentre cohorts to determine its clinical utility for stratifying patients at risk of vaccine failure. These findings suggest that the BAFF/BAFF-R axis may play an important role in vaccine-induced humoral immunity in antibody-deficient patients, warranting further mechanistic investigation.

## Teaser

Patients with antibody deficiencies who failed to respond to SARS-CoV-2 vaccination showed impaired B-cell function, characterised by reduced IgA class-switching, lower overall cell yields in vitro, and diminished BAFF-R expression. While plasma cell differentiation capacity and IGH repertoire diversity were largely preserved, BAFF-R surface expression was consistently lower in non-responders, without evidence of compensatory BAFF pathway activation. These findings suggest that reduced BAFF-R expression reflects a B-cell-intrinsic defect and may represent a promising biomarker for identifying patients at risk of vaccine failure, pending validation in larger cohorts.

## Introduction

B-cells are crucial for humoral immunity, producing antigen-specific antibodies through tightly regulated differentiation pathways. Defects in B-cell differentiation, observed in primary (PAD) and secondary (SAD) antibody deficiencies, can impair immune responses and increase infection susceptibility [1, 2]. PADs, such as common variable immunodeficiency (CVID), are driven by intrinsic immune defects, which are genetically defined in some cases but remain idiopathic in many instances [3]. In contrast, SADs result from external factors, typically malignancies or immunosuppressive therapies that impair B-cell function [3, 4].

The COVID-19 pandemic highlighted the vulnerability of these patients, with approximately one quarter of immunodeficient individuals who contracted SARS-CoV-2 dying during the initial wave [5]. While antibody deficiency is often assumed to preclude vaccine responsiveness, this is not universally the case [6, 7]. Differentiating responders from non-responders within well-characterized antibody-deficient cohorts presents an opportunity to uncover immunological factors necessary for protection, challenging long-held assumptions regarding vaccine failure in this population.

The COVID-19 in patients with antibody deficiency study is a multi-site initiative, encompassing heterogeneous cohorts of both SAD and PAD cases [5–7]. Initial data revealed that nearly a quarter of patients failed to respond to three vaccine doses, with clinical factors such as bronchiectasis, splenomegaly, liver disease, and immunosuppressive therapy potentially contributing to non-response. Despite these insights, a significant gap remains in understanding factors driving vaccine response versus non-response.

The present study aims to characterize B-cell-intrinsic responses using a validated *in vitro* B-cell differentiation model. By identifying defects in B-cell pathways, we seek to uncover factors that distinguish vaccine responders from non-responders, enhancing our understanding of vaccine failure in patients.

Herein, we report that non-responders generated comparable proportions of plasma cells (PC) to responders but had reduced IgA class-switched immunoglobulin *in vitro*. Additionally, we identify reduced B-cell activating factor receptor (BAFF-R) expression as a feature associated with severe acute respiratory syndrome coronavirus 2 (SARS-CoV-2) vaccine non-responsiveness in antibody-deficient patients. These discovery-phase findings support BAFF-R as a candidate biomarker warranting further validation in larger, multicentre cohorts to determine its clinical utility for stratifying patients at risk of vaccine failure.

## Materials and methods

### Sample collection

Peripheral blood mononuclear cell (PBMC) samples were obtained from healthy donors and patients enrolled in the COV-AD study with PAD and SAD after informed consent [5–7]. Samples were collected within 28 days following the third or fourth dose of the SARS-CoV-2 vaccine. Patients were eligible if they were ≥18 years old and either receiving immunoglobulin replacement therapy or had a serum IgG <4 g/L with antibiotic prophylaxis. Diagnoses were assigned using European Society of Immunodeficiencies (ESID) Clinical Working Party criteria. CVID was defined as hypogammaglobulinaemia with reduced serum IgG and IgA and/or IgM, impaired antibody production following vaccination and/or infection, and exclusion of secondary causes. Hypogammaglobulinaemia was defined as persistently reduced IgG (with or without low IgA/IgM) not meeting full ESID criteria for CVID. Specific polysaccharide antibody deficiency (SPAD) was defined as impaired response to polysaccharide vaccines despite normal total immunoglobulin levels. SAD was attributed to defined causes such as rheumatological disease or renal pathology (Table 1).

**Table 1:** Clinical and immunological features of antibody-deficient patients of a cohort of patients enrolled in the COV-AD study (*n* = 49).

Study identifier	Age	Sex	Diagnosis	Immunoglobulin (Product)	Vaccine administered	Post-V2 IGAM ratio	Post-V2 response	Post-V3 IGAM ratio	Post-V3 response	Pre-treatment IgG (g/L)	Trough IgG (g/L)	IgA (g/L)	IgM (g/L)	Lymph (×10^9^/L)	CD3 (×10^9^/L)	CD4 (×10^9^/L)	CD8 (×10^9^/L)	CD19 (×10^9^/L)	CD56 (×10^9^/L)
P1	63	M	CVID	IVIG	AZ	3.71	Yes	6.14	Yes	3.3	9.2	0.9	0.8	0.576	0.51	0.424	0.077	0.039	0.016
P2	56	F	Secondary—HAEM	IVIG	Pfizer	5.53	Yes	20.39	Yes	2.6	7.8	0.01	0.5	0.838	0.761	0.489	0.254	0.01	0.07
P3	34	M	CVID	IVIG	AZ	0.13	No	n/a	n/a	n/a	9.8	n/a	n/a	0.925	0.699	0.362	0.322	0.075	0.123
P4	53	M	Secondary—HAEM	IVIG	AZ	0.31	No	4.16	Yes	0.5	9.1	0.1	0.1	0.953	0.826	0.52	0.271	0.01	0.099
P5	62	M	CVID	IVIG	AZ	1.90	Yes	6.17	Yes	6.1	8.5	0.1	0.5	1.028	0.781	0.289	0.426	0.229	0.01
P6	56	F	Secondary—RHEUM	SCIG	AZ	4.47	Yes	4.56	Yes	4.3	7.4	0.1	0.2	1.704	1.394	0.618	0.756	0.11	0.153
P7	34	F	CVID	IVIG	Pfizer	3.18	Yes	4.12	Yes	n/a	7.2	0.1	1.4	1.392	0.881	0.427	0.375	0.389	n/a
P8	34	F	Secondary—HAEM	None	AZ	0.18	No	0.14	No	0.1	n/a	0.01	0.01	2.1	1.89	0.874	0.809	0.12	0.115
P9	38	F	Secondary—HAEM	None	AZ	0.20	No	0.35	No	1.6	n/a	0	0	1.1	0.692	0.285	0.378	0.144	0.119
P10	69	M	CVID	IVIG	AZ	0.18	No	0.72	No	0.01	9	0.1	0.1	1.313	1.162	0.418	0.378	0.066	0.039
P11	62	M	CVID	SCIG	AZ	3.37	Yes	6.22	Yes	3.5	10	0	0.2	2.4	1.526	0.944	0.41	0.172	0.171
P12	64	F	Secondary—HAEM	IVIG	Pfizer	4.57	Yes	4.98	Yes	5.9	9.9	0.2	0.9	1.125	0.913	0.625	0.271	0.139	0.067
P13	72	F	Hypogammaglobulinaemia	IVIG	AZ	1.61	Yes	6.52	Yes	4.6	6.2	n/a	n/a	0.889	0.678	0.519	0.127	0.079	0.144
P14	78	F	CVID	IVIG	AZ	2.50	Yes	7.45	Yes	3.7	8.6	0.55	0.4	n/a	0.913	0.673	0.214	0.04	0.117
P15	76	F	Secondary—HAEM	IVIG	Pfizer	5.02	Yes	5.90	Yes	7	10.8	1.3	1.7	1.039	0.912	0.669	0.206	0.035	0.074
P16	60	M	CVID	IVIG	Pfizer	0.19	No	0.97	No	5.1	9.1	0.1	1	0.632	0.562	0.336	0.2	0.03	0.023
P17	59	F	Secondary—RHEUM	IVIG	Pfizer	1.30	Yes	1.61	Yes	5.7	10.5	0.5	0.1	0.681	0.335	0.127	0.193	0.003	0.311
P18	67	M	CVID	IVIG	AZ	3.36	Yes	7.12	Yes	n/a	6.6	0.2	0.2	2.241	0.76	0.579	0.155	1.765	1.765
P19	51	M	Secondary—HAEM	IVIG	AZ	1.06	Yes	3.16	Yes	1	3.42	0	0.03	2.14	1.2	0.61	0.43	0.81	0.12
P20	71	F	Secondary—HAEM	SCIG	AZ	4.65	Yes	8.76	Yes	5.09	13.1	1.3	0.7	1.51	0.62	0.37	0.24	0.51	0.37
P21	76	F	Secondary—RHEUM	IVIG	AZ	0.84	No	5.97	Yes	4.46	8.35	3.18	0.52	3.98	2.96	2.24	0.71	0.33	0.68
P22	35	M	Secondary—HAEM	SCIG	Pfizer	0.82	No	4.99	Yes	1.2	9.38	0	1	3.45	2.76	1.34	1.35	0.5	0.18
P23	31	F	CVID	None	AZ	0.29	No	0.74	No	3.55	n/a	0.22	0.26	2.48	2.35	1.34	0.92	0	0.11
P24	66	F	Secondary—HAEM	None	Pfizer	4.27	Yes	5.42	Yes	3.5	n/a	0.48	0.21	1.3	n/a	n/a	n/a	n/a	n/a
P25	24	F	STAT3 Hyper IgE	IVIG	AZ	1.56	Yes	3.67	Yes	n/a	9.9	0.7	0.5	1.739	1.273	0.819	0.435	0.348	0.058
P26	43	F	CVID	IVIG	AZ	1.32	Yes	3.51	Yes	1.3	7.9	0.1	0.3	1.957	1.511	0.67	0.781	0.256	0.199
P27	72	F	Secondary—HAEM	IVIG	AZ	1.92	Yes	5.97	Yes	n/a	6.6	0.2	2	0.253	0.204	0.052	0.153	0.016	0.03
P28	73	F	CVID	IVIG	AZ	16.23	Yes	21.41	Yes	n/a	12.9	0.9	0.4	1.404	1.042	0.707	0.315	0.203	0.119
P29	37	M	CVID	IVIG	AZ	2.81	Yes	5.30	Yes	3.9	7.7	0.2	0.3	0.645	0.485	0.308	0.157	0.102	0.048
P30	26	M	CVID	IVIG	AZ	1.07	Yes	0.83	No	4.2	10.1	0.9	0.6	0.742	0.574	0.384	0.181	0.039	0.083
P31	76	F	Hypogammaglobulinaemia	IVIG	Pfizer	5.25	Yes	6.62	Yes	6.4	10.8	0.9	4	1.496	1.103	0.903	0.181	0.064	0.247
P32	53	F	CVID	IVIG	Pfizer	1.53	Yes	0.37	No	n/a	8.7	0.1	0.1	1.699	1.174	0.609	0.471	0.564	0.035
P33	59	F	Secondary—HAEM	IVIG	Pfizer	0.21	No	0.80	No	n/a	9.8	0.1	0.1	10	1.648	1.367	0.307	10	0.036
P34	53	M	Secondary—RENAL	IVIG	AZ	4.88	Yes	5.17	Yes	0.7	4.9	0.4	0.1	1.524	1.056	0.612	0.452	0.092	0.288
P35	71	F	Hypogammaglobulinaemia	IVIG	AZ	2.31	Yes	4.35	Yes	5.1	9	0.7	0.2	0.641	0.532	0.43	0.1	0.054	0.04
P36	75	M	CVID	IVIG	Pfizer	3.86	Yes	1.34	Yes	n/a	12.9	0.1	0.1	1.8	1.119	0.414	0.675	0.199	0.486
P37	63	F	SPAD	IVIG	Pfizer	5.63	Yes	20.39	Yes	n/a	12.5	0.7	0.3	1.52	0.983	0.574	0.396	0.195	0.273
P38	43	M	CVID	IVIG	AZ	0.31	No	2.18	Yes	n/a	10	0.1	2.7	0.697	0.519	0.253	0.243	0.04	0.1
P39	38	F	Secondary—RHEUM	IVIG	Pfizer	1.65	Yes	0.84	No	5.9	10.9	0.1	0.1	1.328	0.989	0.41	0.521	0.02	0.291
P40	69	F	Secondary—RHEUM	IVIG	Pfizer	1.09	Yes	3.37	Yes	n/a	8.9	1.7	0.5	1.079	0.904	0.475	0.451	0.076	0.101
P41	50	F	CVID	IVIG	Pfizer	2.61	Yes	3.85	Yes	n/a	9.8	0.01	0.1	1.203	0.956	0.685	0.254	0.088	0.135
P42	61	F	Secondary—HAEM	IVIG	AZ	1.59	Yes	2.17	Yes	6.1	9.3	0.4	0.01	0.936	0.63	0.247	0.349	0.129	0.139
P43	74	F	CVID	IVIG	AZ	1.20	Yes	6.35	Yes	5	7.5	1	0.2	1.423	0.859	0.727	0.121	0.279	0.271
P44	59	F	Hypogammaglobulinaemia	IVIG	AZ	2.56	Yes	5.49	Yes	5	10.8	0.5	0.2	1.718	1.244	0.745	0.381	0.297	0.142
P45	33	F	CVID	SCIG	AZ	0.19	No	0.27	No	n/a	8.5	n/a	n/a	0.795	0.639	0.362	0.228	0.043	0.066
P46	70	M	CVID	IVIG	Pfizer	0.59	No	0.55	No	1.19	8.45	0.26	0.17	2.318	1.836	0.358	1.489	0.206	0.273
P47	56	F	CVID	IVIG	AZ	2.63	Yes	5.47	Yes	4.4	8.1	0.49	0.4	n/a	1.182	0.713	0.438	0.141	0.064
P48	56	F	CVID	SCIG	AZ	0.18	No	0.26	No	n/a	13.4	0	0	n/a	0.271	0.241	0.027	0.057	0.021
P49	74	M	Secondary—HAEM	IVIG	AZ	0.67	No	5.39	Yes	1.3	8.4	0.1	1.3	0.452	0.196	0.076	0.113	0.091	0.124

Summary of demographic data, diagnosis, immunoglobulin therapy, COVID-19 vaccine type, and serological responses post-dose 2 (V2) and 3 (V3), measured by anti-spike IgGAM ratio in patients with recoverable B-cells. Pre-treatment IgG, trough immunoglobulin levels (IgG, IgA, and IgM), and lymphocyte subset counts (CD3, CD4, CD8, CD19, and CD56) are also included. Diagnoses include common variable immunodeficiency (CVID), and secondary immunodeficiencies related to haematological (HAEM) or rheumatological (RHEUM) conditions, as well as other rare immunodeficiencies. Data shown for patients P1–P49 represent a subset of the COV-AD cohort included in the analysis. Red denotes vaccine non-responders. Red denotes vaccine non-responders.

All patient samples were cryopreserved prior to analysis, as fresh PBMCs were not available. This approach was supported by in-house optimization and validated using matched fresh and cryopreserved PBMCs from three independent healthy controls (HCs), which showed no discernible differences in terms of phenotype, isotype expression, or cell number (Supplementary Figure 1). This is also consistent with published work [8], reporting minimal effects of cryopreservation on B-cell function. Fresh HC PBMCs were used in the main assay to enable timely processing.

For patient samples, PBMCs were isolated within 24 h of collection using Leucosep tubes pre-filled with Lymphoprep (StemCell Technologies) or, where necessary, Ficoll-Paque PLUS (VWR International). PBMCs were resuspended in freezing medium (90% heat-inactivated FBS and 10% DMSO), aliquoted at 5–10 × 10⁶ cells/ml, and frozen in a CoolCell controlled-rate freezing container at –80°C overnight before transfer to liquid nitrogen (≤ –150°C). Thawing was performed at room temperature until completely thawed, following by washing in Iscove’s Modified Dulbecco’s Medium (Thermo Fisher) with 10% FBS before B-cell differentiation assays. HC PBMCs were processed fresh using comparable Lymphoprep isolation methods.

Details on patient recruitment and sample collection are available in prior reports [5–7]. Ethical approval was granted by London-Dulwich Research Ethics Committee (21/LO/0162) and Leeds East Research Ethics Committee (07/Q1206/47).

### B-cell differentiation

B-cells were isolated from PBMCs using a Miltenyi B-cell isolation kit according to the manufacturer’s protocol. This protocol first depletes non-B-cells using a negative selection cocktail resulting in a total B-cell preparation. For HC samples in direct comparison experiments, the separation of total B-cells was followed by enrichment of naive B-cells via anti-CD27 magnetic bead depletion (Miltenyi Biotec), yielding a predominantly CD19⁺CD27⁻ population as confirmed by flow cytometry.

B-cells were cultured at 2.5 × 10⁵ cells/ml in Iscove’s Modified Dulbecco’s Medium (IMDM) containing 10% FBS. For activation at day 0, cells were stimulated with 20 U/ml human IL-2 (Miltenyi), 50 ng/ml human IL-21 (Peprotech), and 10 μg/ml F(ab′)₂ goat anti-human IgM/IgG/IgA (Stratech), and co-cultured with irradiated murine fibroblasts expressing CD40L (CD40L-L cells). In some experiments, 100 ng/ml BAFF (AdipoGen), the upper range recommended for B-cell activation and differentiation [9], was also included.

On day 3, cells were removed from CD40L-L cells and reseeded in fresh media supplemented with MEM amino acid solution (1:50) and lipid mixture 1 (1:200) (Sigma), containing 20 U/ml IL-2 and 50 ng/ml IL-21. Where indicated, culture media at day 3 also included 100 ng/ml BAFF (AdipoGen). At day 6, cells were reseeded in media supplemented with 10 ng/ml human IL-6 (Peprotech), 10 ng/ml IL-21, and 100 ng/ml APRIL (R&D Systems Biotechne). Cultures were terminated at day 13 to assess the generation of PCs.

### Flow cytometry

Immunophenotyping of B-cells was performed using the following antibodies: CD19-PE (LT19, Thermo Fisher Scientific), CD20-eFluor 540 (2H7, Thermo Fisher Scientific), BAFF-R-BV421 (11C1, BD Biosciences), CD27-FITC (MT271, BD Biosciences), CD38-PE-Cy7 (HB7, BD Biosciences), and CD138-APC (MI15, Miltenyi). B-cells were identified as CD19⁺CD20⁺CD38ˡᵒCD27⁺/⁻CD138⁻; plasmablasts as CD19⁺CD20ˡᵒCD38ʰⁱCD27⁺CD138⁻; and mature PCs as CD19⁺CD20ˡᵒ/⁻CD38ʰⁱCD27⁺CD138⁺. These definitions refer to the proportion of cells expressing the relevant markers. Live/dead discrimination was conducted using Zombie UV (BioLegend). For intracellular immunoglobulin staining, cells were fixed, permeabilized, and stained with IgM-APC (G20-127, BD Biosciences), IgG-FITC (G18-145, BD Biosciences), and IgA-VioBlue (IS11-8E10, Miltenyi).

Cells were stained in MACS buffer (PBS with 0.5% BSA) and analysed on a Beckman Coulter CytoFlex LX flow cytometer. Doublets were excluded based on forward scatter area versus height, and dead cells were excluded using Zombie UV staining. Cell counts were quantified with CountBright™ Absolute Counting Beads (Invitrogen). Data were analysed using FlowJo version 10 (BD Biosciences) and Prism 10 (GraphPad).

### Immunoglobulin secretion

Supernatants were analysed for IgM, IgG, and IgA using enzyme-linked immunosorbent assays (ELISA). ELISAs were performed with Human IgM ELISA Quantitation Set (E80-100), Human IgG ELISA Quantitation Set (E80-104), or Human IgA ELISA Quantitation Set (E80-102) (Bethyl Laboratories Inc.) according to the manufacturer’s instructions.

### RNA analysis

RNA was extracted from cell suspensions using TRIzol (Invitrogen) and sequencing was performed by Novogene (at 20 M reads per sample). Raw sequencing data was quality assessed using FastQC (v0.12.1) and trimmed of adapters using Cutadapt (v5.0). High-quality reads were mapped to GRCh38.p13 reference genome and gene counts were quantified using Salmon (v1.10.3) [10, 11] Differential gene expression analysis was performed using DESeq2 (v1.24.0) in RStudio, and data was visualized using ggplot2 (v3.5.2) [12, 13].

### Immunoglobulin heavy-chain repertoire analysis

Immunoglobulin heavy-chain (IGH) sequences were amplified via a two-step PCR process using BIOMED-2 primers [14]. Indexed libraries were prepared with Nextera reagents and sequenced using Illumina platforms. Raw reads were analysed with MiXCR software and V/J gene combinations were visualized using CIRCOS plots (https://mk.bcgsc.ca/tableviewer/visualize/).

### BAFF quantification

Serum TNFSF13B (BAFF) levels were measured using the SOMAscan^®^ assay (SomaLogic, Inc.) and reported in relative fluorescence units (RFU).

### Statistical analysis

Statistical analyses were performed using Prism 10 (GraphPad), applying one-way ANOVA or Kruskal–Wallis test with Dunn’s multiple comparisons *post hoc* test, or Wilcoxon rank-sum test for pairwise comparisons, as appropriate. Statistical significance was defined as *P* < 0.05 (*P < 0.05; **P < 0.01; ***P < 0.001; ****P < 0.0001).

## Results

### Patient cohort characteristics

For the characterization of B-cells in the context of response to SARS-CoV-2 vaccination amongst antibody-deficient individuals, samples were used from 49 of the patients enrolled in the COV-AD study. Patients selected for *in vitro* differentiation analysis were chosen based on the availability of detailed metadata (Table 1), prioritising patients with confirmed presence of B-cells to assess potential B-cell-intrinsic defects. The selected cohort comprised a heterogeneous group of both SARS-CoV-2 vaccine responding (*n* = 34) and non-responding (*n* = 15) patients with various PAD (*n* = 29) and SAD (*n* = 20). Samples were nationally obtained, and patients were both male and female adults, aged 24–78 (Tables 1 and 2). Previous findings from the COV-AD study primarily define non-responders based on their inadequate serological response following a second dose of either the approved Pfizer BioNTech (162b2) or AstraZeneca (ChAdOx1 nCoV-19) vaccine. Specifically, non-responders had an IgGAM ratio <1.0, measured 1–2 months post-vaccination using anti-spike glycoprotein antibody assays [15]. Non-responders also lacked neutralizing antibody activity and showed poor T-cell responses [6, 7]. Ten naive-enriched B-cell samples from HCs were included in parallel. Naive-enriched B-cells from HCs were used for most experiments, as the memory B-cell compartment was reduced or absent in many patients, although proportions varied across diagnoses (Supplementary Figure 2A). Furthermore, a comparison of total versus naive-only B-cells at day 0 confirmed that variation in memory B-cell frequencies did not account for differences in baseline phenotype or differentiation outcomes (Supplementary Figure 2B–D).

**Table 2:** Characteristics of patients enrolled in the COV-AD study in which total B-cells were successfully isolated.

	Overall	Vaccine responders	Vaccine non-responders
*N*	49	34	15
Primary antibody deficiency, *n*	29	21	8
Secondary antibody deficiency, *n*	20	13	7
Female, *n*	32	25	7
Male, *n*	17	9	8
Age range (years)	24–78	24–78	31–74

Response to SARS-CoV-2 vaccination as determined post-third or -fourth vaccination.

### BAFF increases *in vitro* cell numbers without impacting phenotype

**Figure 1:**
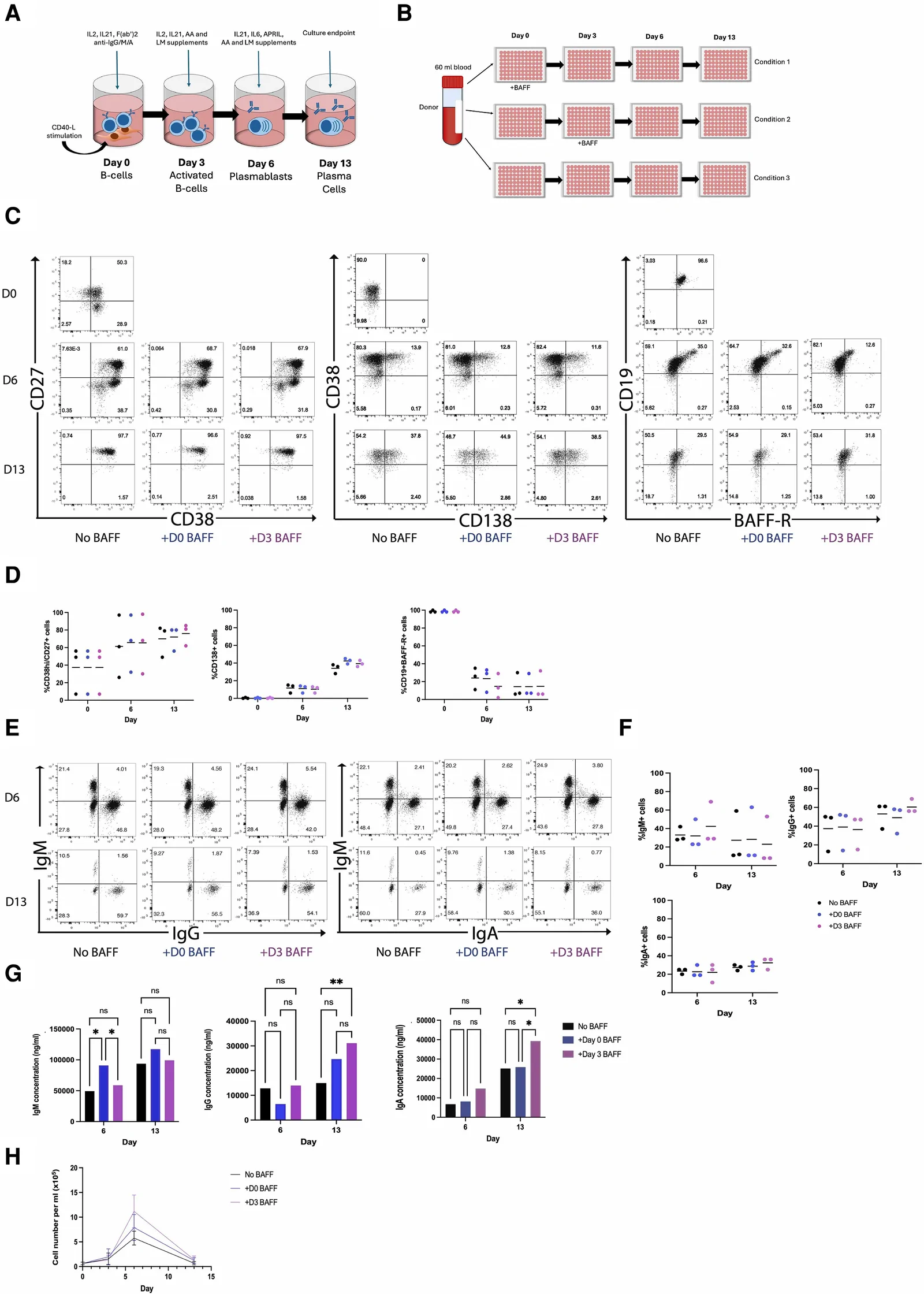
The addition of BAFF to activated total B-cells enhances cell counts without altering phenotype. (a) Schematic of standard *in vitro* B-cell differentiation assay. (b) Experimental setup for the evaluation of the temporal effects of B-cell activation factor (BAFF) on B-cell activation and differentiation. Total B-cells were isolated from a HC and the impact of 100 ng/ml BAFF addition at day 0 versus day 3 of culture, relative to no addition, was assessed. (c) Representative flow cytometry plots showing CD38^hi^CD27⁺, CD38⁺CD138⁺, and CD19⁺BAFF-R⁺ expression following T-dependent stimulation. (d) Relative percentages and averages of CD38^hi^CD27⁺, and CD38⁺CD138⁺ and CD19⁺BAFF-R⁺ populations gated from total B-cells in three independent HCs (*n* = 3) at days 0, 6, and 13 (D0, D6, and D13). Each plot represents the percentage of cells expressing the indicated marker; colours indicate different culture conditions. (e) Intracellular IgM, IgG, and IgA expression at D6 and D13 in a representative HC. (f) Relative percentages and averages of IgM⁺, IgG⁺, and IgA⁺ populations at D6 and D13 in three independent HCs (*n* = 3). (g) Quantification of secreted IgM, IgG, and IgA under different BAFF conditions at D6 and D13. (h) Viable cell counts in three independent HCs (*n* = 3) during differentiation under no BAFF, day 0 BAFF, or day 3 BAFF conditions, shown as mean ± SD.

Initial data from the COV-AD cohort revealed low B-cell numbers in the peripheral blood of patients, likely reflecting a combination of underlying immunodeficiency and sample cryopreservation. This was particularly marked in non-responders, who exhibited pronounced peripheral B-cell lymphopenia on average, despite some individuals falling within the anticipated healthy range of 0.2–0.6 × 10⁹/L (Supplementary Figure 3A). This raised concerns about the feasibility of *in vitro* differentiation, as the minimal B-cell count required for productive differentiation using this system (Fig. 1a) had not been established.

Titration experiments using fresh HC PBMCs demonstrated that CD19⁺ B-cells could survive and undergo differentiation into immunoglobulin-producing CD38^hi^CD27⁺ plasmablasts and CD38^hi^CD138⁺ PCs, even when seeded at reduced input numbers using a constant B-cell to CD40L-fibroblast ratio (Supplementary Figure 3B–E and Supplementary Table 1). While output was proportionally reduced (Supplementary Figure 3F), the phenotypic profile of differentiated cells was maintained, indicating that the assay remained functional despite lower starting cell numbers. However, this optimization was performed on fresh HC samples and may not fully account for the additional impact of cryopreservation on patient-derived PBMCs.

BAFF, a TNF-family cytokine essential for B-cell survival, engages BAFF-R, TACI, and BCMA to modulate B-cell activation and differentiation [16]. Given its role in enhancing B-cell survival *in vitro* [17–19], we hypothesized that BAFF supplementation could increase patient B-cell recovery without altering phenotype, thereby improving assay reproducibility.

To test this, total B-cells from HCs were stimulated under three conditions: (1) BAFF added to pre-activated B-cells at day 0, (2) BAFF added at day 3 following activation, and (3) no BAFF, in keeping with the validated standard protocol [20–22] (Fig. 1b). CD38, CD138, CD27, CD19, and BAFF-R expression were assessed to confirm B-cell differentiation and evaluate BAFF-mediated effects.

At day 0, pre-activated B-cells exhibited a uniform CD19⁺BAFF-R⁺ phenotype. By day 6, BAFF-treated cultures, particularly those with day 3 BAFF addition, retained higher levels of BAFF-R expression, though surface expression was subsequently downregulated as differentiation progressed. Despite donor-specific variability, phenotypic markers and intracellular immunoglobulin expression followed the expected differentiation patterns across HCs (Fig. 1c–f). Secreted immunoglobulin levels were largely comparable (Fig. 1g), although day 3 BAFF addition was associated with modestly higher class-switched immunoglobulin levels at day 13. These findings were recapitulated in naive-enriched B-cell culture (Supplementary Figure 4A–C).

Enumeration of cell numbers demonstrated the expected B-cell-expanding effect of BAFF, with BAFF-treated cultures exhibiting higher cell recovery compared with untreated cultures. Notably, the greatest expansion was observed in cultures supplemented with BAFF at day 3, a result that was consistently replicated across all three samples (Fig. 1h). This finding was further validated in naive-enriched B-cell cultures (Supplementary Figure 4D), reinforcing the reproducibility and efficacy of 100 ng/ml BAFF addition at this time point. Accordingly, BAFF supplementation at day 3 was applied to patient-derived samples to optimize cell recovery in subsequent experiments.

### Patient samples generate plasmablasts and PCs *in vitro*

Investigating the inherent differentiation capacity of B-cells is essential to understanding whether non-responders fail to produce sufficient PCs, possibly contributing to poor vaccine responses. Flow cytometry phenotyping was conducted on all 49 samples using our *in vitro* differentiation model with data from two representative samples per group (HCs, responders, and non-responders) shown in detail (Table 1).

**Figure 2:**
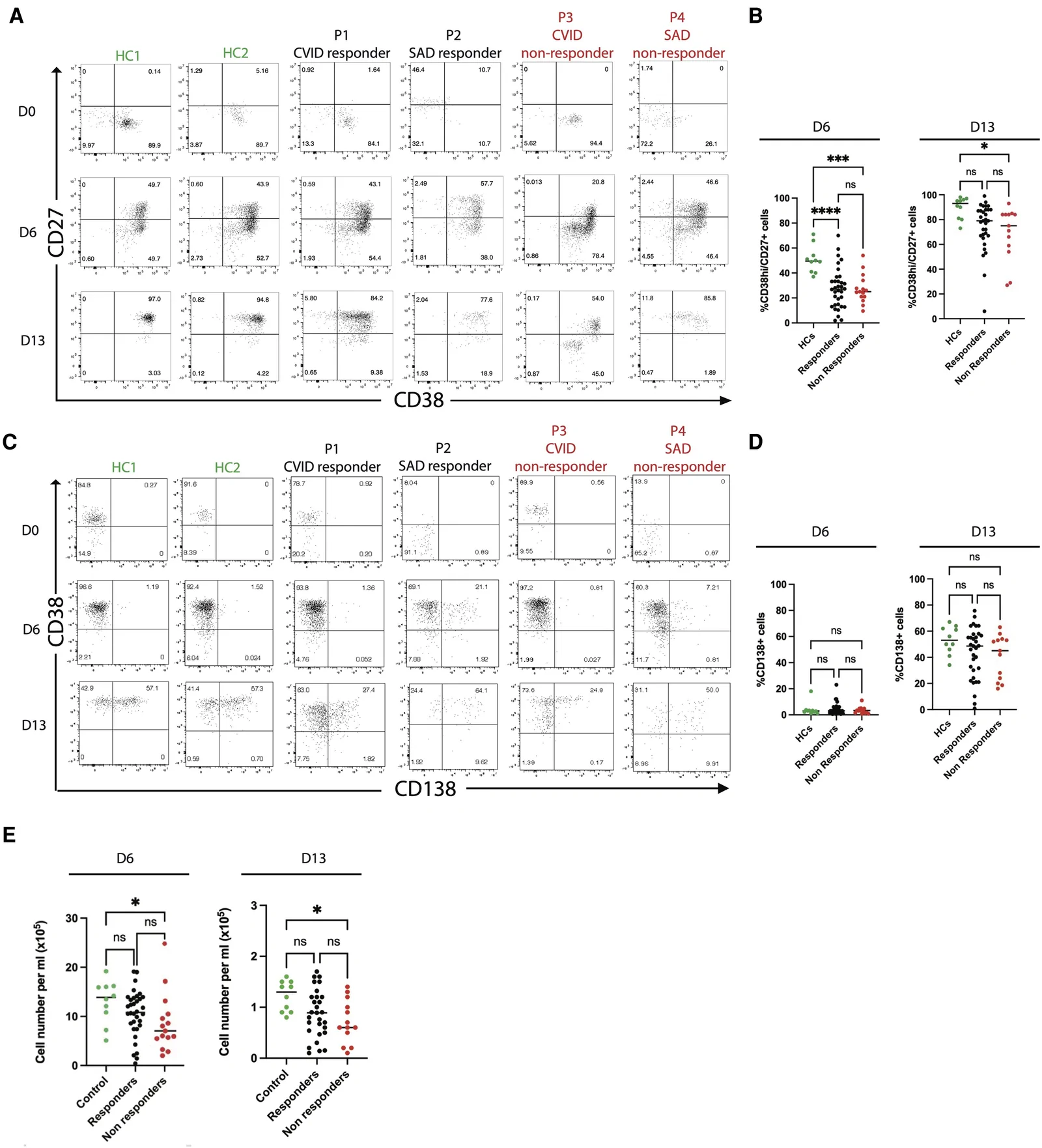
Vaccine-responding and non-responding antibody-deficient patient total B-cells are capable of generating plasma cells *in vitro*. (a) Representative example of naive B-cells isolated from HCs; CVID vaccine-responder; SAD vaccine-responder; CVID vaccine non-responder and SAD vaccine non-responder. B-cells were assessed at days 0, 6, and 13 for plasmablast and plasma cell generation by the gain of CD27 and CD38 after T-dependent stimulation conditions. (b) Quantification of the percentage of cells expressing CD38^hi^CD27+ at day 6 and day 13. (c) Representative examples of naive B-cells isolated from HCs; common variable immunodeficiency (CVID) vaccine-responder; secondary antibody deficient (SAD) vaccine-responder; CVID vaccine non-responder and SAD vaccine non-responder. B-cells were assessed at days 0, 6, and 13 for plasma cell differentiation by the gain of CD38 and CD138 after stimulation with T-dependent stimulation conditions. (d) Quantification of the percentage of cells expressing CD138 at day 6 and day 13. HC naive B-cells, *n* = 10; Vaccine-responding patient B-cells, *n* = 34; Vaccine non-responding patient B-cells, *n* = 15. Statistical significance was determined using one-way ANOVA or Kruskal–Wallis test with Dunn’s multiple comparisons (*P < 0.0001, ***P < 0.001, *P < 0.01, *P < 0.05**). (e) Total cell numbers per millilitre at day 6 and day 13 in HCs (*n* = 10), vaccine-responding patients (*n* = 34), and vaccine non-responding patients (*n* = 15). Statistical significance was determined using one-way ANOVA or Kruskal–Wallis test with Dunn’s multiple comparisons (*P < 0.0001, ***P < 0.001, *P < 0.01, *P < 0.05**).

The representative HCs displayed the expected gradual increase in CD27⁺ cells, reaching >95% CD38^hi^CD27⁺ by day 13, which encompasses both plasmablasts and PCs. This pattern was also observed in representative patient samples, albeit to varying extents (Fig. 2a). While all representative samples showed an overall increase in CD38^hi^CD27⁺ populations, the majority exhibited lower frequencies of these cells compared with controls at day 6 in both responders and non-responders. By day 13, a similar trend was observed; however, statistical significance persisted only between controls and non-responders. The magnitude of this difference was smaller than at day 6, and considerable inter-patient variability was evident in both patient groups (Fig. 2b).

Across the cohort, no significant differences in the percentages of CD138^+^ PCs were observed between controls, vaccine responders, and non-responders (Fig. 2c and d). Although representative CVID patients showed reduced PC generation compared with controls, this was not consistently linked to vaccine response status (Fig. 2c).

Collectively, phenotypic data examining PC/plasmablast generation show that, despite differences indicating reduced differentiation capacities across all COV-AD samples, both vaccine response groups were able to generate similar proportions of CD38^hi^CD27^+^ plasmablasts and CD38^hi^CD27^+^CD138^+^ PCs.

Nevertheless, patient cultures showed reduced recovery overall, with non-responders exhibiting significantly lower cell counts compared with controls at both time points when antibody-secreting cells are generated (Fig. 2e).

### *In vitro*-generated patient ASCs produce reduced levels of class-switched immunoglobulins

The loss or failure of immunoglobulin function, particularly IgG, is a hallmark of antibody deficiency [1]. Given evidence linking impaired immunoglobulin class-switching to diminished SARS-CoV-2 vaccine responses [23, 24], we investigated whether these findings extended to our cohort by assessing intracellular and secreted IgM, IgG, and IgA expression.

**Figure 3:**
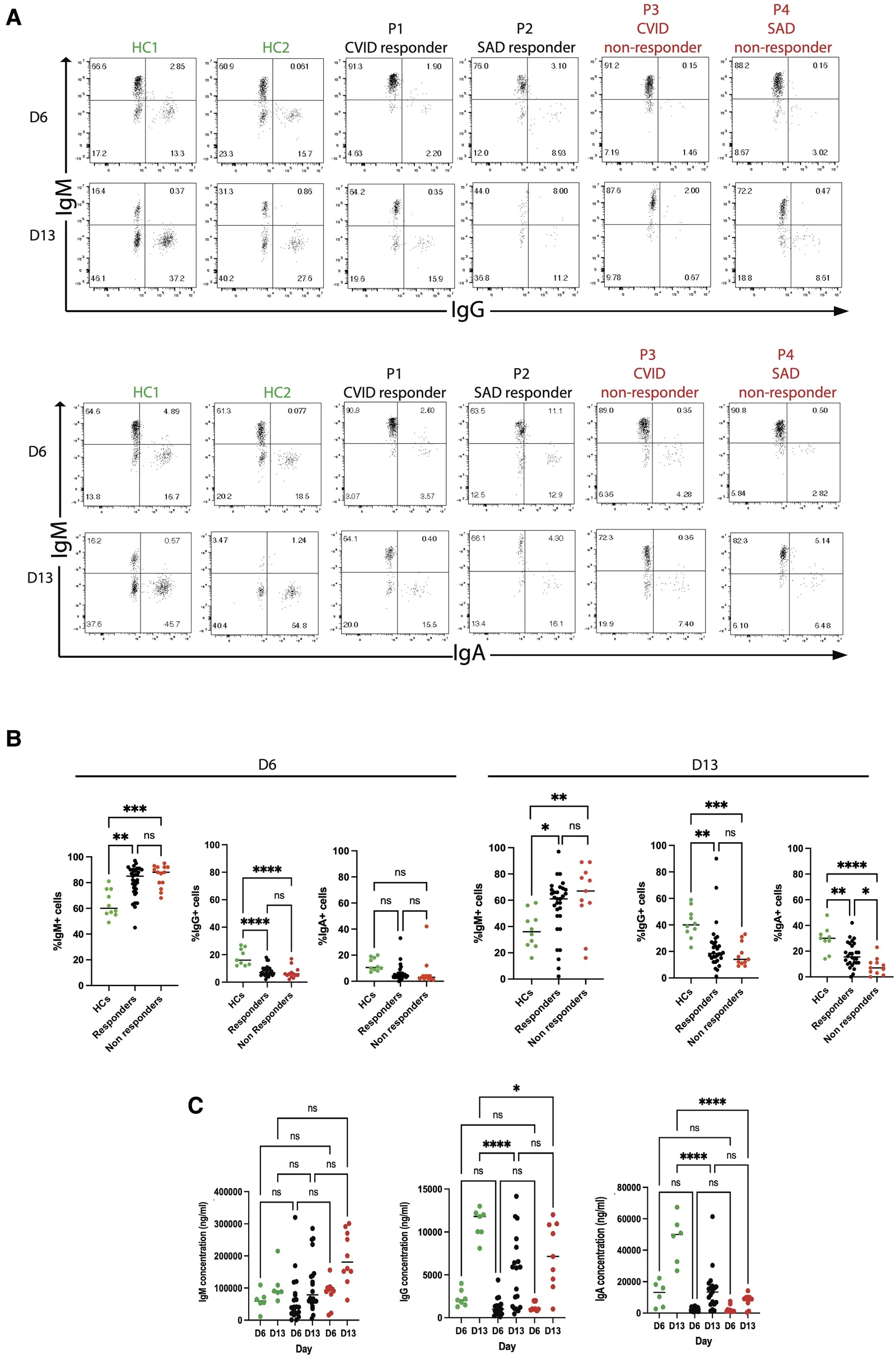
Vaccine-responding and non-responding antibody-deficient patients show no discernible difference in immunoglobulin expression. (a) Representative examples of intracellular IgM, IgG, and IgA expression in naive B-cells isolated from healthy controls (HCs); CVID vaccine-responding; SAD vaccine-responding; SAD vaccine non-responding and CVID vaccine non-responding patients at D6 and D13. (b) Assessment of the percentage of cells expressing IgM, IgG, and IgA at D6 and D13. HC naive B-cells, *n* = 10; vaccine-responding patient B-cells, *n* = 33; vaccine non-responding patient B-cells, *n* = 14. (c) Secreted IgM, IgG, and IgA expression in HCs, vaccine-responding and vaccine non-responding patients. Statistical significance was determined using one-way ANOVA or Kruskal–Wallis test with Dunn’s multiple comparisons (*P < 0.0001, ***P < 0.001, *P < 0.01, *P < 0.05**).

Representative controls exhibited the expected intracellular immunoglobulin profile, with high IgM at day 6 that declined by day 13, alongside increased IgG and IgA (Fig. 3a). In contrast, patient samples exhibited persistently elevated IgM and reduced IgG and IgA expression at both time points. Both responders and non-responders had significantly higher IgM and lower IgA expression than controls; for IgG, both patient groups showed lower expression than controls, but only IgA was significantly reduced in non-responders compared with responders (Fig. 3b), a trend not accounted for by baseline memory B-cell frequencies, which were comparable between groups (S2C and D). This suggests that reduced induction of IgA *in vitro*, rather than with clinical IgA deficiency itself, may be associated with poor SARS-CoV-2 vaccine response in antibody-deficient patients [25].

Trends in immunoglobulin secretion largely mirrored intracellular findings. Both patient groups had significantly lower IgG and IgA secretion than controls by day 13. While non-responders showed a tendency towards increased IgM, no statistically significant differences in secreted IgM, IgG, or IgA were observed between responders and non-responders (Fig. 3c).

### Patient samples generate polyclonal PCs *in vitro*

The human immune repertoire relies on diverse V(D)J segment combinations within the immunoglobulin heavy chain (IGH) locus to generate antibodies against a wide range of pathogens. Given this, vaccine non-responders may exhibit alterations in IGH V–J segment usage, potentially limiting repertoire diversity. To investigate this, IGH V–J gene segment combinations were compared between selected vaccine-responding and non-responding patients (P5–10, Table 1) using sequences obtained from day 13 *in vitro*-differentiated cultures of total B-cells isolated from patients.

**Figure 4:**
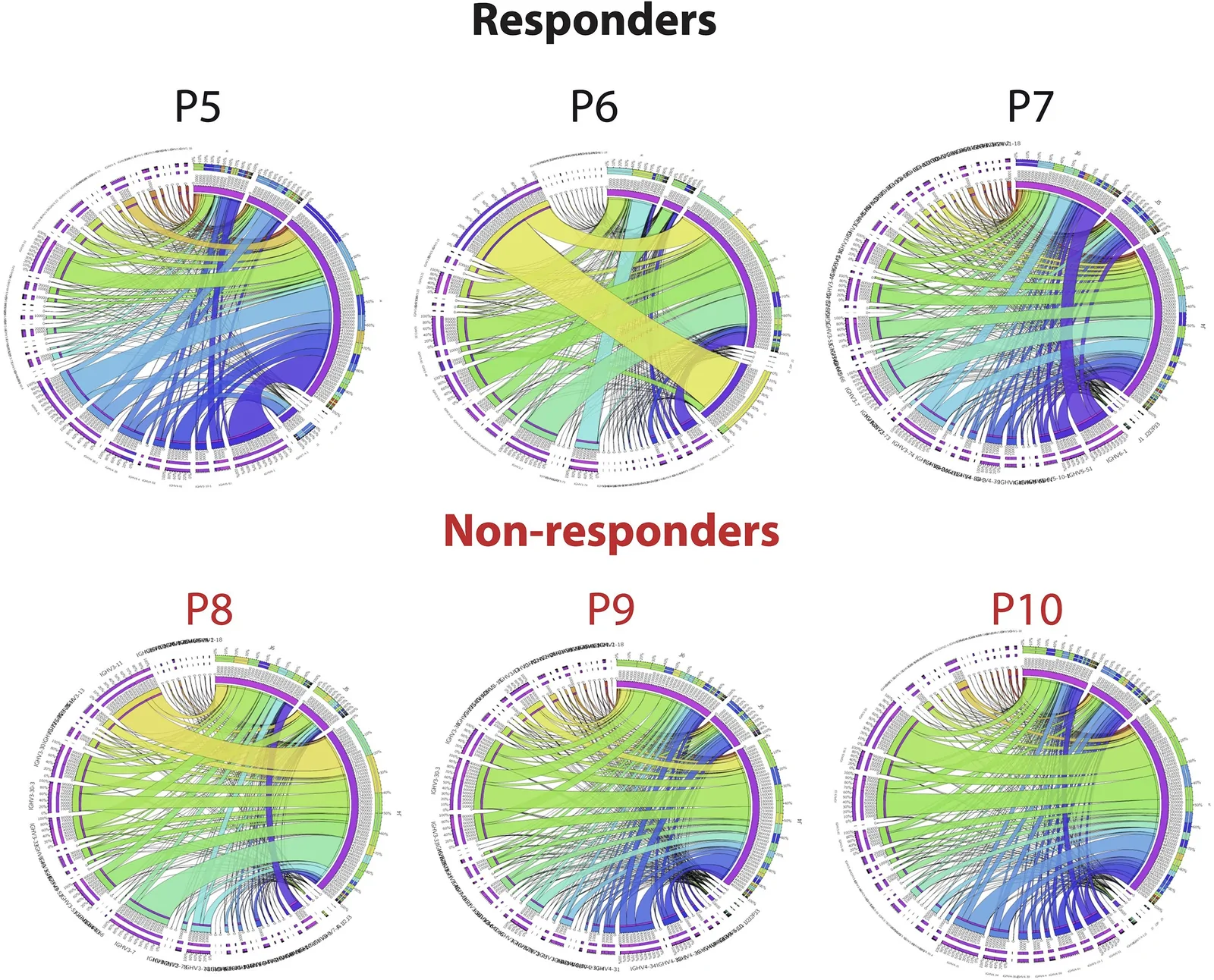
Vaccine-responding and non-responding antibody-deficient patients show no discernible difference in V–J recombination. V and J gene usage in COV-AD patients (P5–P10) from BCR sequences obtained from RNA extracted at D13 of *in vitro* differentiation. Samples include SARS-CoV-2 vaccine-responders (*n* = 3) and non-responders (*n* = 3). Arc lengths represent the relative frequency of V or J gene usage, and ribbon widths depict clonotype connection frequencies. Colours indicate distinct clonotypes, illustrating diversity and clonality within each sample.

Consistent with previous reports of preserved IGH repertoire diversity in antibody-deficient patients [26], all patient samples exhibited broadly distributed V-J pairings (Fig. 4a), indicating retained immunoglobulin gene usage regardless of vaccine response. Whilst no major differences were observed between responders and non-responders, P18 showed highly restricted V-J pairings, likely reflecting an underlying diagnosis of chronic lymphocytic leukaemia (CLL) that was retrospectively identified during analysis (Supplementary Figure 5). This underscores the utility of the system in flagging patients with markedly impaired B-cell function, including those with underlying haematological malignancy.

To further explore whether IGH repertoire characteristics could distinguish vaccine responders from non-responders, we examined the variable heavy chain complementarity-determining region 3 (CDR3) length and net charge, as these features can influence antigen recognition and may reflect repertoire immaturity or autoreactivity [27]. CDR3 lengths and charges appeared elevated in some non-responders compared with the rest of the cohort; however, there was considerable variability amongst all patients, and the small sample size was insufficient for meaningful statistical analysis (Supplementary Figure 6A and 6B).

These findings suggest that IGH diversity, both at the level of V-J recombination and basic CDR3 physicochemical properties, is generally preserved in vaccine non-responders. Functional defects in these patients are therefore more likely to result from downstream mechanisms, such as signalling defects or impaired maturation, rather than from constraints in repertoire generation [28].

### Vaccine non-responders have significantly reduced or absent Baff-R expression on B-cells

**Figure 5:**
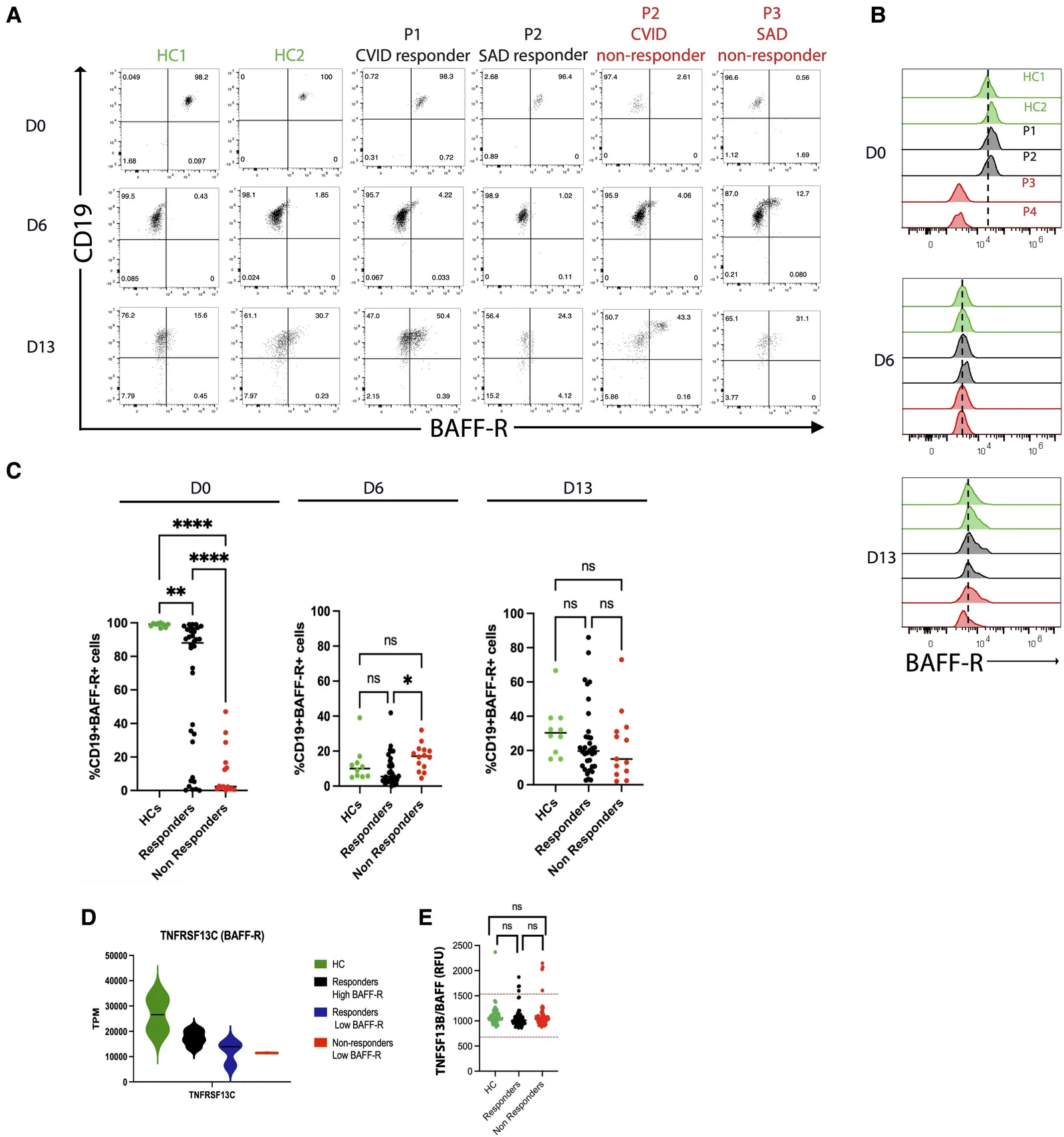
Vaccine non-responders have significantly reduced or absent BAFF-R expression in B-cells. (a) Representative plots showing BAFF-R and CD19 expression at days 0, 6, and 13 under T-dependent stimulation in naive B-cells isolated from healthy controls (HCs), CVID vaccine-responders, SAD vaccine-responders, CVID vaccine non-responders, and SAD vaccine non-responders. (b) Histograms illustrating BAFF-R expression across time points in representative individuals. (c) Quantification of CD19⁺BAFF-R⁺ B-cells at day 0, day 6, and day 13. Naive B-cells isolated from HCs, *n* = 10; vaccine-responders, *n* = 34; vaccine non-responders, *n* = 15. (d) Gene expression of *TNFRSF13C* (encoding BAFF-R) in naive B-cells isolated from HCs (*n* = 2), vaccine-responders with high (*n* = 5) and low (*n* = 3) surface BAFF-R expression at day 0, and vaccine non-responders (*n* = 2) shown in transcripts per million (TPM). (e) Serum TNFSF13B/BAFF levels in HCs, vaccine-responders, and vaccine non-responders measured in relative fluorescence units (RFU). Statistical significance was determined using one-way ANOVA or Kruskal–Wallis test with Dunn’s multiple comparisons (*P < 0.0001, ***P < 0.001, *P < 0.01, *P < 0.05**).

To further explore whether non-responders might have alterations in key external regulators of the B-cell lineage, we assessed the proportion of CD19⁺ B-cells expressing BAFF-R [28–30]. In line with expected patterns [31], naive B-cells from HCs exhibited a high proportion of BAFF-R+ cells at day 0. This proportion declined by day 6 and remained low at day 13, consistent with differentiation-associated downregulation (Fig. 5a).

In contrast, most vaccine non-responders displayed a lower proportion of BAFF-R⁺ total B-cells at day 0 compared with HCs and the majority of responders. By day 6, BAFF-R expression had decreased in all groups, consistent with differentiation-associated downregulation, and non-responders maintained a lower proportion of BAFF-R⁺ B-cells throughout culture. A subset of responders also exhibited low BAFF-R expression at day 0 (Fig. 5a and b), an observation that did not correlate with borderline IgGAM values (Table 1) or with consistent differences in cell phenotype, frequency, or antibody secretion during *in vitro* differentiation.

Given the differences in BAFF-R⁺ proportions at day 0, RNA was collected from matched samples at this time point to determine whether transcriptional dysregulation of *TNFRSF13C*, the gene encoding BAFF-R, contributed to the phenotype. Selected samples represented both CVID and SAD patients, spanning vaccine responders and non-responders with a range of BAFF-R expression. Low BAFF-R expression was defined as ≤50% of the HC median. RNA sequencing revealed reduced *TNFRSF13C* transcript abundance in both responders and non-responders with low BAFF-R surface expression, suggesting that diminished transcription may contribute to the phenotype (Fig. 5d). However, TNFRSF13C expression was variable within each group, including among HCs, indicating that post-transcriptional or other regulatory mechanisms may also be involved.

Reduced BAFF-R expression has been described in antibody deficiencies, particularly CVID, where it can inversely correlate with serum BAFF levels and associate with increased TACI expression [32]. We therefore hypothesized that reduced BAFF-R expression in non-responders might be linked to elevated serum BAFF, leading to increased expression of alternative receptors TACI and BCMA. To explore this, BAFF-R, TACI, and BCMA expression was assessed at days 0, 6, and 13. Although reductions in BAFF-R expression were confirmed, TACI and BCMA followed expected differentiation-associated patterns in all groups [32], with no evidence of compensatory upregulation in patients with low BAFF-R expression at day 0 (Supplementary Figure 7).

Finally, to test whether impaired BAFF-R expression was associated with elevated serum BAFF, serum BAFF concentrations (TNFSF13B) were measured. No significant differences were observed between groups (Fig. 5e), suggesting that systemically elevated BAFF is unlikely to account for reduced BAFF-R expression in this cohort.

## Discussion

The vulnerability of antibody-deficient patients to severe infections, notably COVID-19, highlights the critical need to better understand the immune dysfunctions underlying these conditions. Despite vaccination efforts, many antibody-deficient individuals remain at heightened risk of poor outcomes due to impaired capacity to generate adequate immune responses. This issue remains especially pertinent in the context of COVID-19 and the emergence of new variants, as well as other infectious diseases that continue to threaten immunocompromised populations.

This study provides novel insights into B-cell-intrinsic features associated with vaccine non-responsiveness, with a particular focus on BAFF-R expression. Using an optimized *in vitro* B-cell differentiation model [20–22], we found that non-responders exhibited significantly reduced total cell numbers compared with HCs and reduced IgA class-switched immunoglobulin production *in vitro* compared with HCs and responders. The most consistent immunophenotypic difference was a lower proportion of BAFF-R⁺ B-cells at baseline in non-responders, with similar patterns observed in a subset of responders.

Impaired BAFF-R expression has previously been associated with heightened disease activity in immunocompromised individuals, including those with CVID [32–35]. Reduced BAFF-R availability has been linked to elevated circulating BAFF levels and compensatory upregulation of alternative receptors such as TACI and BCMA [32, 36–38]. However, this was not observed in our cohort. Patients with diminished BAFF-R^+^ proportions did not show accompanying increases in TACI or BCMA, but did exhibit relatively lower transcript levels of *TNFRSF13C*. In addition to mRNA abundance, surface receptor availability may be influenced by post-transcriptional regulation, mRNA stability, receptor shedding, or altered protein trafficking [39–41].

These observations support a model in which diminished BAFF-R expression reflects a multifactorial interplay of genetic, epigenetic, and environmental factors [41–43], rather than a single pathway defect. While our findings demonstrate an association between reduced BAFF-R⁺ proportions and vaccine non-responsiveness, causality cannot be inferred from this study. Nevertheless, BAFF-R remains a promising candidate biomarker that warrants further evaluation for its potential to stratify patients at higher risk of poor vaccine responses, potentially informing the use of additional vaccine doses or passive immunization approaches.

Our results align with recent evidence implicating the BAFF/BAFF-R axis in vaccine efficacy. For example, Faliti *et al*. [44] reported that anti-BAFF therapy correlated with reduced vaccine-induced antibody responses, supporting the view that BAFF signalling is important for B-cell survival and differentiation. The association between reduced BAFF-R expression and altered differentiation trajectories in our model suggests that impaired signalling, whether from reduced receptor expression or downstream defects, may contribute to suboptimal humoral immunity in antibody-deficient individuals. However, the exact mechanistic links between BAFF-R dysfunction and impaired vaccine responses remain incompletely defined.

Future work should focus on delineating the molecular mechanisms regulating BAFF-R expression and function, and on understanding how these influence short- and long-term vaccine efficacy. Larger, multicentre studies that include a wide range of primary and secondary immunodeficiencies will be essential to determine whether specific conditions are disproportionately affected and to validate these findings. Such studies should use fresh samples with a more homogeneous starting B-cell population to minimize technical variability and strengthen mechanistic insights. Extending these investigations to vaccines against other pathogens will help establish whether the observed associations reflect broader principles of impaired vaccine responsiveness.

By integrating mechanistic analysis with the evaluation of clinically relevant candidate biomarkers, this work lays the groundwork for developing more personalized vaccine strategies that could improve immune outcomes and guide targeted interventions for vulnerable patient groups.

## Supplementary Material

uxag004_Supplementary_Data

## Data Availability

The data that support the findings of this study are available from the corresponding author upon reasonable request. Processed and raw RNA-seq data will be publicly available at time of publication; Gene Expression Omnibus accession number: GSE298709.

## Abbreviations

BAFF-R: B-cell activating factor receptor
BAFF: B-cell activating factor
APRIL: a proliferation-inducing ligand
PC: plasma cell
ASC: antibody-secreting cell
PBMC: peripheral blood mononuclear cell
HC: healthy control
PAD: primary antibody deficiency
SAD: secondary antibody deficiency
SPAD: specific polysaccharide antibody deficiency
CVID: common variable immunodeficiency
COV-AD: COVID-19 in antibody deficiency
SARS-CoV-2: severe acute respiratory syndrome coronavirus 2
IGH: immunoglobulin heavy chain
BCR: B-cell receptor
TACI: transmembrane activator and CAML interactor
BCMA: B-cell maturation antigen
CLL: chronic lymphocytic leukaemia
CDR3: complementarity-determining region 3
ESID: European Society for Immunodeficiencies
IMDM: Iscove’s Modified Dulbecco’s Medium
FBS: foetal bovine serum
DMSO: dimethyl sulfoxide
ELISA: enzyme-linked immunosorbent assay
RNA-seq: RNA sequencing
TPM: transcripts per million
RFU: relative fluorescence units
TNFRSF13C: tumour necrosis factor receptor superfamily member 13C
TNFSF13B: tumour necrosis factor ligand superfamily member 13B

## References

[1] Duraisingham SS, Buckland M, Dempster J, Lorenzo L, Grigoriadou S, Longhurst HJ. Primary vs. secondary antibody deficiency: clinical features and infection outcomes of immunoglobulin replacement. PLoS ONE 2014, 9, e100324. 24971644 10.1371/journal.pone.0100324 PMC4074074

[2] Tangye SG, Al-Herz W, Bousfiha A, Chatila T, Cunningham-Rundles C, Etzioni A, et al Human inborn errors of immunity: 2019 update on the classification from the international union of immunological societies expert committee. J Clin Immunol 2020, 40, 24–64. 31953710 10.1007/s10875-019-00737-x PMC7082301

[3] Herriot R, Sewell WA. Antibody deficiency. J Clin Pathol 2008, 61, 994–1000. 18755724 10.1136/jcp.2007.051177

[4] Dhalla F, Lucas M, Schuh A, Bhole M, Jain R, Patel SY, et al Antibody deficiency secondary to chronic lymphocytic leukemia: should patients be treated with prophylactic replacement immunoglobulin? J Clin Immunol 2014, 34, 277–82. 24557494 10.1007/s10875-014-9995-5

[5] Shields A, Anantharachagan A, Arumugakani G, Baker KF, Bahal S, Baxendale H, et al Outcomes following SARS-CoV-2 infection in patients with primary and secondary immunodeficiency in the UK. Clin Exp Immunol 2022, 209, 247–58. 35641155 10.1093/cei/uxac008 PMC8807296

[6] Shields A, Faustini S, Hill HJ, Al-Taei S, Tanner C, Walder S, et al SARS-CoV-2 vaccine responses in individuals with antibody deficiency: findings from the COV-AD study. J Clin Immunol 2022, 42, 923–34. 35420363 10.1007/s10875-022-01231-7 PMC9008380

[7] Shields A, Faustini S, Hill HJ, Al-Taei S, Tanner C, Ashford F, et al Increased seroprevalence and improved antibody responses following third primary SARS-CoV-2 immunisation: an update from the COV-AD study. Front Immunol 2022, 13, 912571. 35720400 10.3389/fimmu.2022.912571 PMC9201027

[8] Hermansen J, Tjønnfjord G, Munthe L, Taskén K, Skånland S. Cryopreservation of primary B cells minimally influences their signaling responses. Nat Sci Rep 2018, 8, 17651. 10.1038/s41598-018-36121-9 PMC6281576 30518828

[9] Thien M, Phan TG, Gardam S, Amesbury M, Basten A, Mackay F, et al Excess BAFF rescues self-reactive B cells from peripheral deletion and allows them to enter forbidden follicular and marginal Zone Niches. Immunity 2004, 20, 785–98. 15189742 10.1016/j.immuni.2004.05.010

[10] Martin M . Cutadapt removes adapter sequences from high-throughput sequencing reads. EMBnet J 2011, 19, 10–11.

[11] Patro R, Duggal G, Love M, Irzarry R, Kingsford C. Salmon provides fast and bias-aware quantification of transcript expression. Nat Methods 2017, 14, 417–9. 28263959 10.1038/nmeth.4197 PMC5600148

[12] Wickham H . Ggplot2: Elegant Graphics for Data Analysis, 2nd ed. Verlag (NY): Springer Publishers, 2016.

[13] Love MI, Huber W, Anders S. Moderated estimation of fold change and dispersion for RNA-Seq data with DESeq2. Genome Biol 2014, 15, 550. 25516281 10.1186/s13059-014-0550-8 PMC4302049

[14] Van Dongen JJM, Langerak AW, Brüggemann M, Evans PAS, Hummel M, Lavender FL, et al Design and standardization of PCR primers and protocols for detection of clonal immunoglobulin and T-cell receptor gene recombinations in suspect lymphoproliferations: Report of the BIOMED-2 Concerted Action BMH4-CT98-3936. Leukemia 2003, 17, 2257–317. 14671650 10.1038/sj.leu.2403202

[15] Faustini S, Jossi S, Perez-Toledo M, Shields A, Allen J, Watanabe Y, et al Development of a high-sensitivity ELISA detecting IgG, IgA and IgM antibodies to the SARS-CoV-2 spike glycoprotein in serum and saliva. Immunology 2021, 164, 135–47. 33932228 10.1111/imm.13349 PMC8242512

[16] Schneider P . The role of APRIL and BAFF in lymphocyte activation. Curr Opin Immunol 2005, 17, 282–9. 15886118 10.1016/j.coi.2005.04.005

[17] Rolink AG, Tschopp J, Schneider P, Melchers F. Baff is a survival and maturation factor for mouse B cells. Eur J Immunol 2024, 32, 2004–10. 10.1002/1521-4141(200207)32:7<2004::AID-IMMU2004>3.0.CO;2-5 12115621

[18] Avery DT, Kalled SL, Ellyard JI, Ambrose C, Bixler SA, Thien M, et al Baff selectively enhances the survival of plasmablasts generated from human memory B cells. J Clin Invest 2003, 112, 286–97. 12865416 10.1172/JCI18025 PMC164292

[19] Marsman C, Verhoeven D, Koers J, Rispens T, Brinke A, van Ham SM, et al Optimized protocols for in-vitro T-cell-dependent and T-cell-independent activation for B-cell differentiation studies using limited cells. Front Immunol 2022, 13, 815449. 35844625 10.3389/fimmu.2022.815449 PMC9278277

[20] Cocco M, Stephenson S, Care MA, Newton D, Barnes NA, Davison A, et al In vitro generation of long-lived human plasma cells. J Immunol 2012, 189, 5773–85. 23162129 10.4049/jimmunol.1103720

[21] Stephenson S, Care MA, Zougman FI, Westhead A, Doody DR, Tooze MG, et al Growth factor-like gene regulation is separable from survival and maturation in antibody-secreting cells. J Immunol 2019, 202, 1287–300. 30642980 10.4049/jimmunol.1801407 PMC6360259

[22] Stephenson S, Care MA, Doody GM, Tooze RM. April drives a coordinated but diverse response as a foundation for plasma cell longevity. J Immunol 2022, 209, 926–37. 36130130 10.4049/jimmunol.2100623 PMC7613700

[23] Montamat G, Meehan CE, Bradford HF, Yıldırım R, Guimarães F, Johnson M, et al Reduced response to SARS-CoV-2 vaccination is associated with impaired immunoglobulin class switch recombination in SLE patients. Clin Exp Immunol 2024, 219, uxae119. 10.1093/cei/uxae119 PMC11773804 39658056

[24] Nakagama Y, Candray K, Kaku N, Komase Y, Rodriguez-Funes MV, Dominguez R, et al Antibody avidity maturation following recovery from infection or the booster vaccination grants breadth of SARS-CoV-2 neutralizing capacity. J Infect Dis 2023, 227, 780–7. 36546706 10.1093/infdis/jiac492 PMC10044078

[25] Parry H, McIlroy G, Bruton R, Ali M, Stephens C, Damery S, et al Antibody responses after first and second covid-19 vaccination in patients with chronic lymphocytic leukaemia. Blood Cancer J 2021, 11, 136. 34330895 10.1038/s41408-021-00528-x PMC8323747

[26] Ghraichy M, Galson JD, Kelly DF, Trück J. B-cell receptor repertoire sequencing in patients with primary immunodeficiency: a review. Immunology 2018, 153, 145–60. 29140551 10.1111/imm.12865 PMC5765381

[27] Bashford-Rogers R, Smith K, Thomas D. Antibody repertoire analysis in polygenic autoimmune diseases. Immunology 2018, 155, 3–17. 29574826 10.1111/imm.12927 PMC6099162

[28] Darce JR, Arendt BK, Wu X, Jelinek DF. Regulated expression of BAFF-binding receptors during human B cell differentiation. J Immunol 2007, 179, 7276–86. 18025170 10.4049/jimmunol.179.11.7276

[29] Wang K, Wei G, Liu D. Cd19: a biomarker for B cell development, lymphoma diagnosis and therapy. Exp Hematol Oncol 2012, 1, 36. 23210908 10.1186/2162-3619-1-36 PMC3520838

[30] Lau AWY, Turner VM, Bourne K, Hermes JR, Chan TD, Brink R. Baffr controls early memory B cell responses but is dispensable for germinal center function. J Exp Med 2021, 218, e20191167. 33119033 10.1084/jem.20191167 PMC7604765

[31] Smulski CR, Eibel H. Baff and BAFF-receptor in B cell selection and survival. Front Immunol 2018, 9, 2285. 30349534 10.3389/fimmu.2018.02285 PMC6186824

[32] Barbosa RR, Silva SL, Silva SP, Melo AC, Conceição Pereira-Santos M, Barata JT, et al Reduced BAFF-R and increased TACI expression in common variable immunodeficiency. J Clin Immunol 2014, 34, 573–83. 24809296 10.1007/s10875-014-0047-y

[33] Warnatz K, Salzer U, Rizzi M, Fischer B, Gutenberger S, Böhm J, et al B-cell activating factor receptor deficiency is associated with an adult-onset antibody deficiency syndrome in humans. Proc Natl Acad Sci U S A 2009, 106, 13945–50. 19666484 10.1073/pnas.0903543106 PMC2722504

[34] Sellam J, Miceli-Richard C, Gottenberg J, Ittah M, Lavie F, Lacabaratz C, et al Decreased B cell activating factor receptor expression on peripheral lymphocytes associated with increased disease activity in primary sjogren’s syndrome and systemic lupus erythematosus. Ann Rheum Dis 2007, 66, 790–7. 17185325 10.1136/ard.2006.065656 PMC1954659

[35] Block V, Sevdali E, Recher M, Abolhassani H, Hammarstrom, Smulski CR, et al CVID-associated B cell activating factor receptor variants change receptor oligomerization, ligand binding, and signaling responses. J Clin Immunol 2023, 43, 391–405. 36308663 10.1007/s10875-022-01378-3 PMC9616699

[36] Matharu K, Zarember KA, Marciano BE, Kuhns DB, Spalding C, Garofalo M, et al B-cell activating factor (BAFF) is elevated in chronic granulomatous disease. Clin Immunol 2013, 148, 258–264. 23773925 10.1016/j.clim.2013.05.007 PMC3774275

[37] Jacobs HM, Thouvenel CD, Leach S, Arkatkar T, Metzler G, Scharping NE, et al Cutting edge: BAFF promotes autoantibody production via TACI-dependent activation of transitional B cells. J Immunol 2016, 196, 3525–31. 27022196 10.4049/jimmunol.1600017 PMC4868625

[38] Pallikkuth S, Kanthikeel SP, Silva SY, Fischl M, Pahwa R, Pahwa S. Innate immune defects correlate with failure of antibody responses to H1N1/09 vaccine in HIV-infected patients. J Allergy Clin Immunol 2011, 128, 1279–85. 21752440 10.1016/j.jaci.2011.05.033 PMC3229646

[39] Smulski CR, Kury P, Seidel LM, Staiger HS, Edinger A, Willen L, et al BAFF- and TACI-dependent processing of BAFFR by ADAM proteases regulates the survival of B cells. Cell Rep 2017, 18, 2189–202. 28249164 10.1016/j.celrep.2017.02.005

[40] Mihalcik SA, Tschumper RC, Jelinek DF. Transcriptional and post-transcriptional mechanisms of BAFF-receptor dysregulation in human B lineage malignancies. Cell Cycle 2010, 9, 4884–9. 21099364 10.4161/cc.9.24.14156 PMC3047811

[41] Gibney ER, Nolan CM. Epigenetics and gene expression. Heredity (Edinb) 2010, 105, 4–13. 20461105 10.1038/hdy.2010.54

[42] Hedrich CM, Tsokos GC. Epigenetic mechanisms in systemic lupus erythematosus and other autoimmune diseases. Trends Mol Med 2011, 17, 714–24. 21885342 10.1016/j.molmed.2011.07.005 PMC3225699

[43] Martínez-Cano J, Campos-Sánchez E, Cobaleda C. Epigenetic priming in immunodeficiencies. Front Cell Dev Biol 2019, 7, 125. 31355198 10.3389/fcell.2019.00125 PMC6635466

[44] Faliti CE, Van TTP, Anam FA, Cheedarla N, Williams ME, Mishra AK, et al Disease-associated B-cells and immune endotypes shape adaptive immune responses to SARS-CoV-2 mRNA vaccination in human SLE. Nat Immunol 2025, 26, 131–45. 39533072 10.1038/s41590-024-02010-9 PMC11695260

