# Supplementary material for: BAFF-R expression as a potential biomarker associated with COVID-19 vaccine non-responsiveness in antibody-deficient patients
Source: Clin Exp Immunol. 2026 Jan 20;220(1):uxag004. doi: 10.1093/cei/uxag004 (PMC13512619; doi:10.1093/cei/uxag004)
Supplement: Supplementary file 1 [file uxag004_supplementary_data.zip › ST1_tables.docx]

# Supplementary Table 1

## A. PBMCs

|  | 15 ml | 10 ml | 5 ml |
| --- | --- | --- | --- |
| HC1 | 2.46 × 10⁵ | 1.6 × 10⁵ | 0.85 × 10⁵ |
| HC2 | 2 × 10⁵ | 1.55 × 10⁵ | 1.1 × 10⁵ |
| HC3 | 3.7 × 10⁵ | 2.4 × 10⁵ | 1.15 × 10⁵ |

## B. B-cells

|  | 15 ml | 10 ml | 5 ml |
| --- | --- | --- | --- |
| HC1 | 1.35 × 10⁵ | 0.35 × 10⁵ | 0.25 × 10⁵ |
| HC2 | 1.05 × 10⁵ | 1.35 × 10⁵ | 0.8 × 10⁵ |
| HC3 | 3.5 × 10⁵ | 1.3 × 10⁵ | 1.0 × 10⁵ |
