# Supplementary material for: BAFF-R expression as a potential biomarker associated with COVID-19 vaccine non-responsiveness in antibody-deficient patients
Source: Clin Exp Immunol. 2026 Jan 20;220(1):uxag004. doi: 10.1093/cei/uxag004 (PMC13512619; doi:10.1093/cei/uxag004)
Supplement: Supplementary file 1 [file uxag004_supplementary_data.zip › SupFig1.pdf]

S1

A

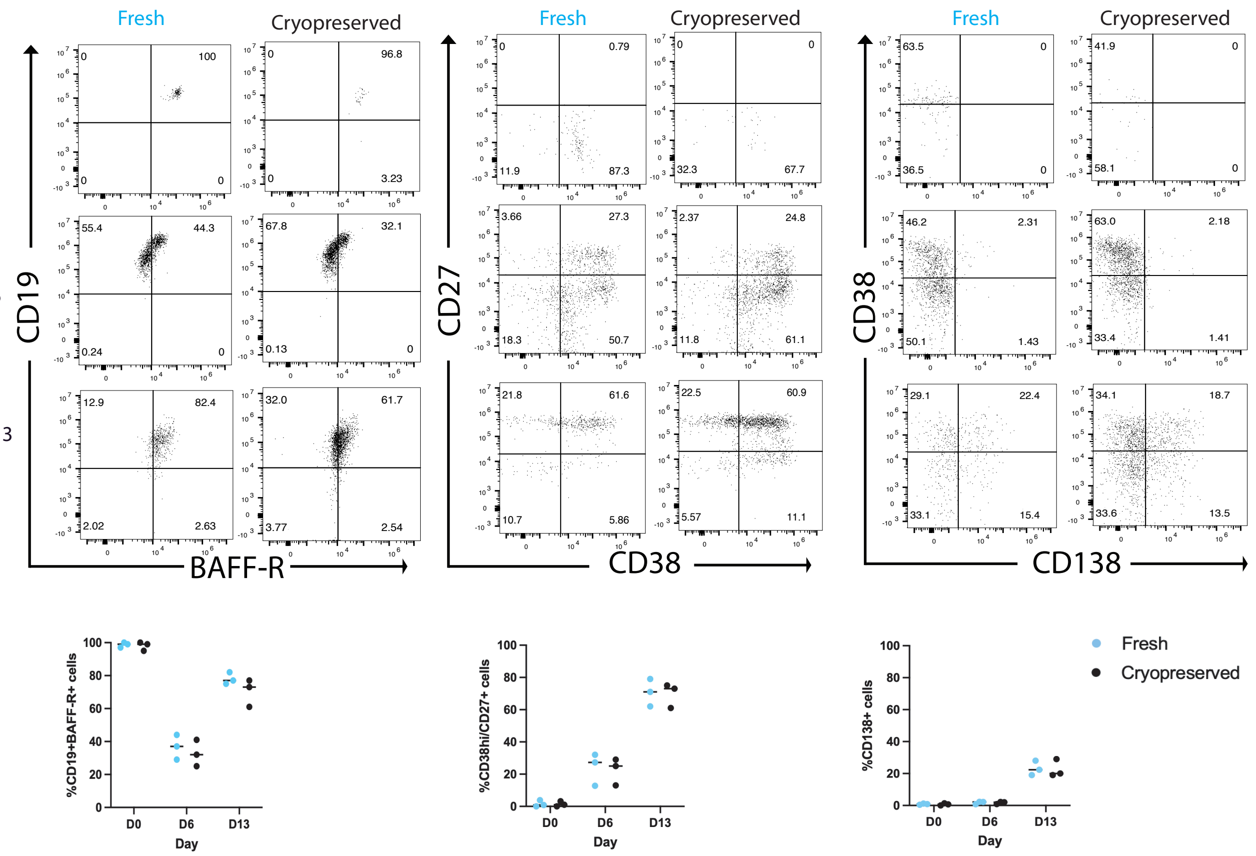
Fresh
Cryopreserved
D0
D6
D13
CD19
BAFF-R
CD27
CD38
CD138
%CD19+BAFF-R+ cells
%CD38hi/CD27+ cells
%CD138+ cells
Day
Fresh
Cryopreserved

B

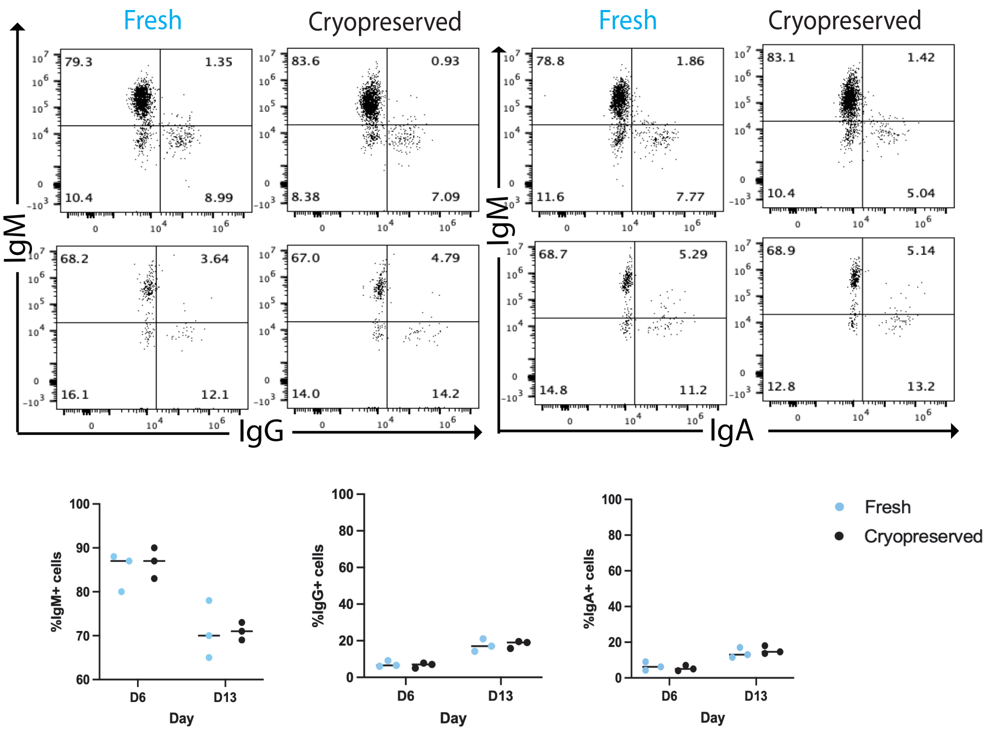
Fresh
Cryopreserved
D6
D13
IgM
IgG
IgA
%IgM+ cells
%IgG+ cells
%IgA+ cells
Day
Fresh
Cryopreserved

C

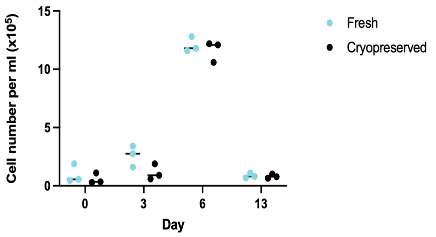
Cell number per ml (x10⁵)
Day
Fresh
Cryopreserved
