# Supplementary material for: BAFF-R expression as a potential biomarker associated with COVID-19 vaccine non-responsiveness in antibody-deficient patients
Source: Clin Exp Immunol. 2026 Jan 20;220(1):uxag004. doi: 10.1093/cei/uxag004 (PMC13512619; doi:10.1093/cei/uxag004)
Supplement: Supplementary file 1 [file uxag004_supplementary_data.zip › SupFig2.pdf]

S2

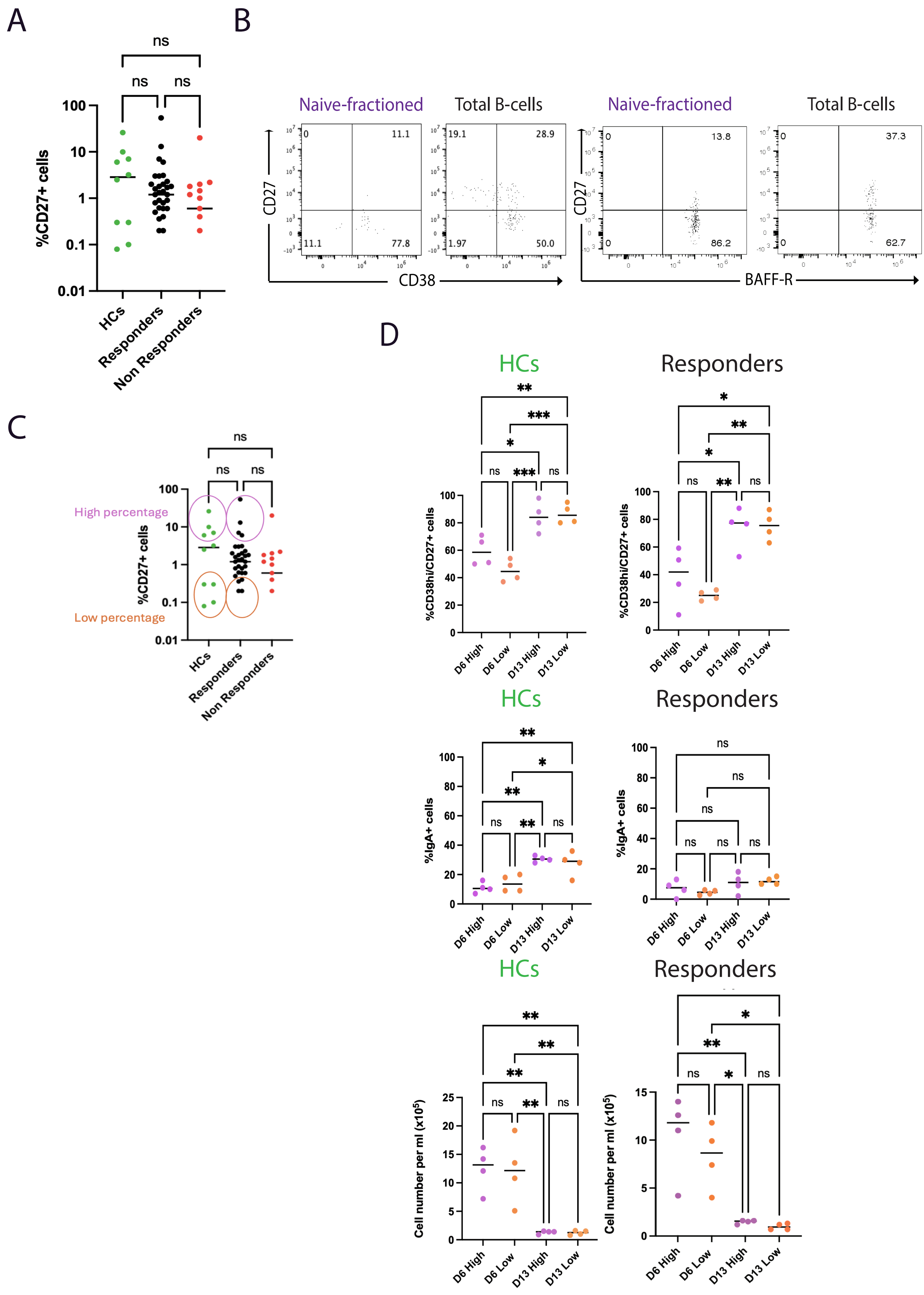

A
B
C
D
ns
%CD27+ cells
HCs
Responders
Non Responders
Naive-fractioned
Total B-cells
CD27
CD38
BAFF-R
11.1
77.8
19.1
28.9
1.97
50.0
13.8
86.2
37.3
62.7
High percentage
Low percentage
%CD38hi/CD27+ cells
%IgA+ cells
Cell number per ml (x10^5)
D6 High
D6 Low
D13 High
D13 Low
