# Supplementary material for: BAFF-R expression as a potential biomarker associated with COVID-19 vaccine non-responsiveness in antibody-deficient patients
Source: Clin Exp Immunol. 2026 Jan 20;220(1):uxag004. doi: 10.1093/cei/uxag004 (PMC13512619; doi:10.1093/cei/uxag004)
Supplement: Supplementary file 1 [file uxag004_supplementary_data.zip › SupFig4.pdf]

S4

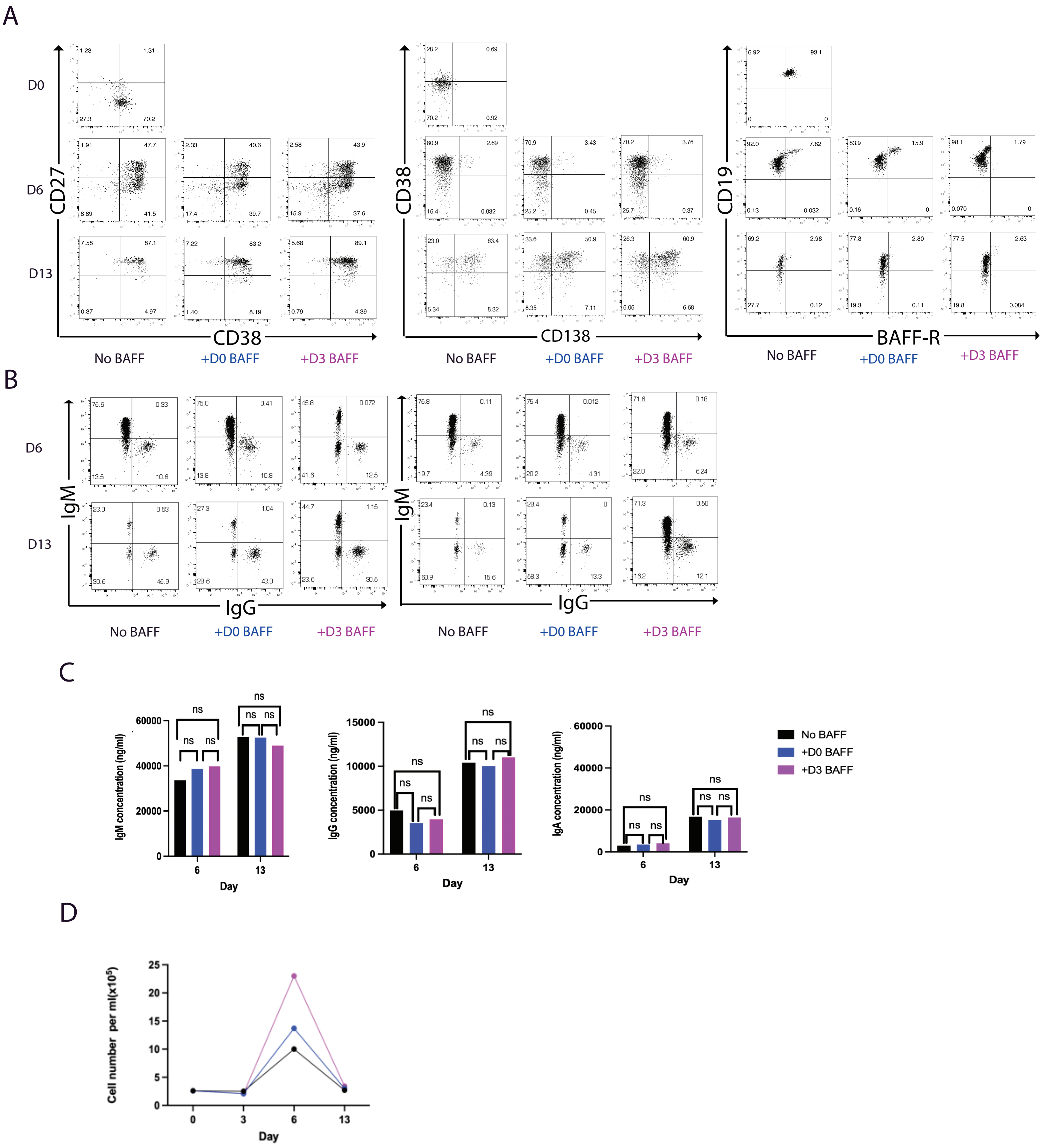
A
D0
D6
D13
CD27
CD38
CD138
CD19
BAFF-R
No BAFF
+D0 BAFF
+D3 BAFF
B
D6
D13
IgM
IgG
C
IgM concentration (ng/ml)
IgG concentration (ng/ml)
IgA concentration (ng/ml)
ns
Day
No BAFF
+D0 BAFF
+D3 BAFF
D
Cell number per ml(x10^5)
Day
