# Supplementary material for: BAFF-R expression as a potential biomarker associated with COVID-19 vaccine non-responsiveness in antibody-deficient patients
Source: Clin Exp Immunol. 2026 Jan 20;220(1):uxag004. doi: 10.1093/cei/uxag004 (PMC13512619; doi:10.1093/cei/uxag004)
Supplement: Supplementary file 1 [file uxag004_supplementary_data.zip › SupFig6.pdf]

S6

A

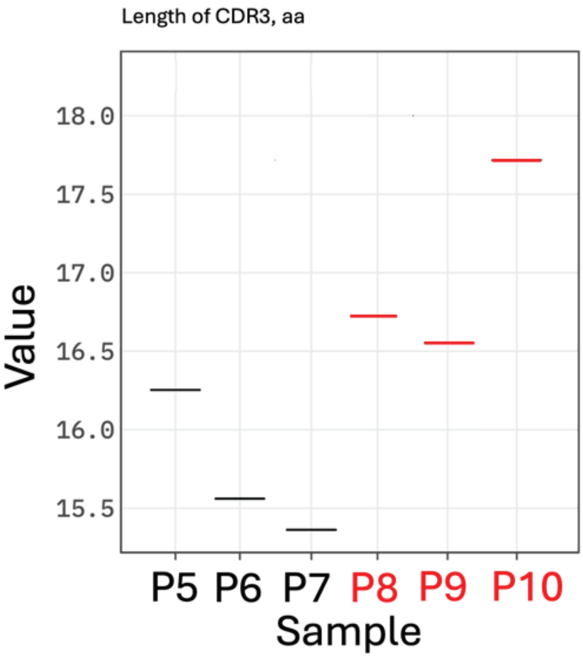
Length of CDR3, aa
18.0
17.5
17.0
16.5
16.0
15.5
Value
P5
P6
P7
P8
P9
P10
Sample

B

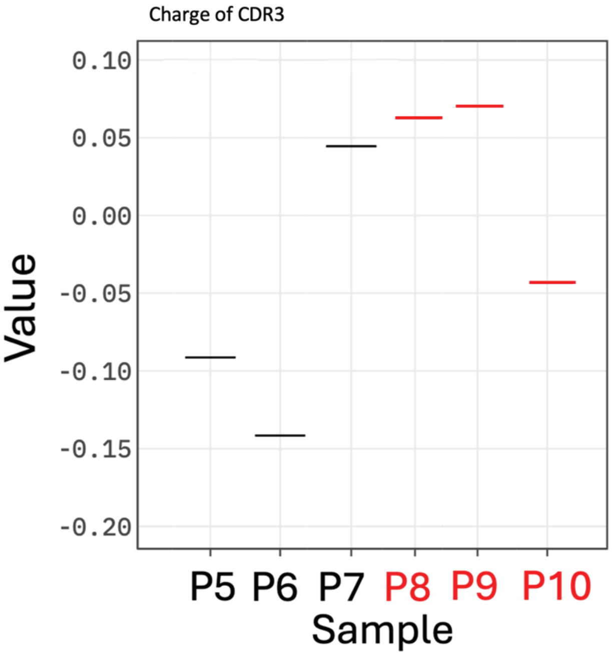
Charge of CDR3
0.10
0.05
0.00
-0.05
-0.10
-0.15
-0.20
Value
P5
P6
P7
P8
P9
P10
Sample
