# Supplementary material for: BAFF-R expression as a potential biomarker associated with COVID-19 vaccine non-responsiveness in antibody-deficient patients
Source: Clin Exp Immunol. 2026 Jan 20;220(1):uxag004. doi: 10.1093/cei/uxag004 (PMC13512619; doi:10.1093/cei/uxag004)
Supplement: Supplementary file 1 [file uxag004_supplementary_data.zip › Supplementary_figure_legends.docx]

**Supplementary Tables and Figures**

### **Supplementary Figure 1: Fresh and cryopreserved PBMCs show no discernible differences in B-cell phenotype, differentiation or expansion outcomes. (A)** Comparison of BAFF-R, CD27, CD38, and CD138 expression on naïve-isolated B-cells from matched fresh and cryopreserved PBMCs at day 0, day 6, and day 13 of culture. Representative flow cytometry plots are shown alongside quantification of the percentage of BAFF-R⁺, CD27⁺, CD38⁺, and CD138⁺ cells for each donor (n = 3). **(B)** Analysis of IgM⁺, IgG⁺, and IgA⁺ B-cell differentiation at day 6 and day 13 for the same fresh and cryopreserved samples. Representative plots and corresponding percentages of IgM⁺, IgG⁺, and IgA⁺ cells are shown (n = 3). (**C**) Total viable cell number measured across donors at day 0, day 6 and day 13 showing no notable difference in cell numbers between fresh and cryopreserved cultures (n=3).

**Supplementary Figure 2: Baseline CD27⁺ frequency does not predict plasma cell features during *in vitro* B-cell differentiation. (A)** Assessment of the percentage of cells expressing CD27+ at day 0 (D0) following purification from PBMCs using non-B cell depletion for patient samples or additional anti-CD27 beads for HCs. Data are displayed on a logarithmic scale. HC naïve B-cells, n=10; vaccine-responding patient B-cells, n=34; vaccine non-responding patient B-cells, n=15. **(B)** Representative flow cytometry plots comparing CD27 and CD38 or BAFF-R expression in naïve-fractioned versus total B-cells from a HC*.* (**C**) Four donors containing the highest percentage and four containing the lowest percentage CD27+ cells from either the HC or responder groups in part A are highlighted (purple = high CD27⁺, orange = low CD27⁺) and subsequently compared in panel D. The non-responder group did not contain sufficient samples with equivalent percentages so were not included. (**D**) Differentiation outcomes for CD27-high vs CD27-low donors analysed separately for HCs (left panels) and responders (right panels)**.** Frequencies of CD38⁺CD27⁺ plasmablasts and IgA⁺ class-switched cells were quantified at day 6 and day 13, along with total viable cell number per ml. Statistical comparisons were performed using one-way ANOVA or Kruskal-Wallis with Dunn’s multiple comparisons (*p<0.05, **p<0.01, ***p<0.001, ****p<0.0001; ns = not significant*).

**Supplementary Figure 3: B-cell recovery and assay performance at limiting input cell numbers (A**) CD19⁺ B-cell counts in peripheral blood mononuclear cells (PBMCs) from the COV-AD cohort, stratified by vaccine response status. Anticipated B-cell counts in healthy samples are typically in the range of 0.2–0.6 × 10⁹/L.**(B)** Whole blood from a representative healthy control (HC) was split into 15 ml, 10 ml, and 5 ml samples. Total B-cells were isolated and cultured under T cell-dependent (TD) conditions. Samples were analysed at days 0, 6, and 13 to assess the impact of reduced input on B-cell differentiation. **(C)** Relative percentages and averages of CD38^hi^CD27⁺, CD138⁺ and CD19+CD20+ populations gated from total B-cells in three independent healthy controls (HCs, n=3) at days 0, 6, and 13 (D0, D6 and D13). Each plot represents the percentage of cells expressing the indicated marker; colours indicate different culture conditions **(D)** Relative percentages and averages of IgM⁺, IgG⁺, and IgA⁺ populations at D6 and D13 in three independent HCs (n=3). **(E)** Quantification of secreted IgM, IgG, and IgA under different conditions at D6 and D13. **(F)** Viable cell counts in three independent HCs (n=3) during differentiation under the different conditions, shown as mean ± SD.

**Supplementary Table 1: Cell counts.** Starting PBMC (**A**) and B-cell (**B**) numbers per ml in three different healthy controls (HC1, HC2 and HC3) from either 15 ml, 10 ml or 5 ml fresh blood.

**Supplementary Figure 4: The addition of BAFF to activated naïve-fractioned B-cells enhances cell counts without altering phenotype**. **(A)** Representative flow cytometry plots showing CD38^hi^CD27⁺, CD38⁺CD138⁺and CD19⁺BAFF-R⁺ expression following T-dependent stimulation at days 0, 6 and 13 (D0, D6 and D13) in naïve B-cells isolated from healthy control (HC) B-cells. **(B)** Intracellular IgM, IgG, and IgA expression at D6 and D13 in a representative HC.(C**)** Quantification of secreted IgM, IgG, and IgA under different BAFF conditions at D6 and D13. **(D)** Viable cell counts during differentiation in the representative control under no BAFF, day 0 BAFF, or day 3 BAFF conditions. Statistical significance was determined using one-way ANOVA or Kruskal-Wallis test with Dunn’s multiple comparisons (******p<0.0001, ***p<0.001, *p<0.01, *p<0.05**).*

**Supplementary Figure 5: BCR repertoire analysis of a COV-AD SARS-CoV-2 vaccine-responding patient (P18) with CVID and concurrent CLL.** Circos plot illustrating the dominant unique clonotype detected at D13 of *in vitro* differentiation. Arc lengths represent the relative usage of V and J gene segments; ribbon widths indicate clonotype connections.

**Supplementary Figure 6**: **CDR3 length and charge in vaccine-responding and non-responding patients.** (A) Mean CDR3 length (amino acids) from immunoglobulin heavy chain (IGH) repertoire analysis at day 13 in selected vaccine-responding (black) and non-responding (red) patients. (B) Mean CDR3 net charge for the same individuals. Each point represents the average value for all unique sequences within an individual. Groups were compared using a Wilcoxon rank-sum test, with no significant differences observed (length: p = –1; charge: p = –1).

**Supplementary Figure 7: Reduced or absent BAFF-R expression is not accompanied by compensatory alterations in TACI or BCMA expression.** Flow cytometric analysis of **BAFF-R (B-cell activating factor receptor), TACI (transmembrane activator and CAML interactor), and BCMA (B-cell maturation antigen)** on isolated B-cells from healthy controls (HCs, green), vaccine responders (grey/black), and vaccine non-responders (red) at day 0, day 6, and day 13 of culture.
